# Proteolytic Processing of Neuregulin 2

**DOI:** 10.1007/s12035-019-01846-9

**Published:** 2019-12-14

**Authors:** Maria Czarnek, Joanna Bereta

**Affiliations:** Department of Cell Biochemistry, Faculty of Biochemistry, Biophysics and Biotechnology, Jagiellonian University in Kraków, Gronostajowa 7, 30-387 Kraków, Poland

**Keywords:** Neuregulins, Neuregulin 2, ADAM10, BACE, BACE2, Shedding

## Abstract

Neuregulin 2 (NRG2) belongs to the EGF family of growth factors. Most of this family members require proteolytic cleavage to liberate their ectodomains capable of binding and activating their cognate ErbB receptors. To date, most of the studies investigating proteolytic processing of neuregulins focused on NRG1, which was shown to undergo ectodomain shedding by several ADAM proteases and BACE1 and the remaining fragment was further cleaved by γ-secretase. Recently, NRG2 attracted more attention due to its role in the neurogenesis and modulation of behaviors associated with psychiatric disorders. In this study, we used genetic engineering methods to identify proteases involved in proteolytic processing of murine NRG2. Using non-neuronal cell lines as well as cultures of primary hippocampal neurons, we demonstrated that the major proteases responsible for releasing NRG2 ectodomain are ADAM10 and BACE2. Co-expression of NRG2 and BACE2 in neurons of certain brain structures including medulla oblongata and cerebellar deep nuclei was confirmed via immunohistochemical staining. The cleavage of NRG2 by ADAM10 or BACE2 generates a C-terminal fragment that serves as a substrate for γ-secretase. We also showed that murine NRG2 is subject to post-translational modifications, substantial glycosylation of its extracellular part, and phosphorylation of the cytoplasmic tail.

**Electronic supplementary material:**

The online version of this article (10.1007/s12035-019-01846-9) contains supplementary material, which is available to authorized users.

## Background

Neuregulin 2 (NRG2) is produced as a type I-oriented transmembrane protein. Of the four NRG genes identified (denoted as NRG1–4), NRG1 and NRG2 share the greatest homology. Like all NRGs, NRG2 has an EGF-like domain located in its extracellular portion; this domain is responsible for binding and activation of the ErbB receptors. Similarly to some NRG1 isoforms, NRG2 also possesses a single Ig-like domain, which may mediate interactions with extracellular matrix proteoglycans. Although both NRG1 and NRG2 bind to the same receptors, ErbB3 and ErbB4 [1, 2], their roles are not interchangeable and knockout (KO) animals show non-overlapping phenotypes. It might be explained by distinct sites of expression of NRG1 and NRG2, different affinities for their cognate ErbB receptors, and triggering different cellular responses [1–3]. Busfield et al. reported that the expression of NRG2 is confined to specific regions of the brain (cerebellum, olfactory bulb, and hippocampal dentate gyrus), non-overlapping with NRG1 expression pattern [4]. However, recent study regarding NRG2 expression in adult mouse brain, which utilized more sensitive methods, suggests that NRG2 expression is more ubiquitous than reported previously [5]. NRG1 seems to be essential during development, because *Nrg1* genetic deletion leads to embryonic lethality due to heart abnormalities [6] that resemble those observed in *Erbb2* and *Erbb4* null mice [7, 8]. Mice lacking NRG1 also display several defects in nervous system development [6, 9]. Unlike *Nrg1*-KO mice, *Nrg2*-KO mice are viable and do not display severe phenotypical abnormalities apart from post-birth growth retardation [5, 10]. Nonetheless, the impaired growth rate is compensated over the course of four months and *Nrg2*-KO mice become indistinguishable from control mice [10]. Alteration of NRG1 expression or genetic polymorphism in *Nrg1* gene are linked to the pathophysiology of schizophrenia [11, 12]. Single nucleotide polymorphism analysis and association studies also pointed to a genome region encompassing NRG2 locus to be associated with the vulnerability for neuropsychiatric diseases [13–17]. A recent study shows that NRG2-KO mice develop dopamine disbalance similar to that observed in schizophrenia and behave abnormally in several behavioral tests [5], again implying a role of NRG2 in the modulation of behavior implicated in psychiatric disorders.

Most members of the EGF family of growth factors rely on proteolytic cleavage to release soluble, ErbB signaling-competent ectodomains. Currently little is known about NRG2 processing and function. NRG1 is a substrate for shedding by several metalloproteases from the ADAM family and the beta-secretase BACE1, which belongs to aspartyl proteases [18–21]. By using a broad-spectrum metalloprotease inhibitor GM6001, Vullhorst et al. have shown that the ectodomain of rat NRG2 is shed by one or more enzymes of a metalloprotease family [22].

Here, we sought to elucidate which proteases are responsible for proteolytic processing of murine NRG2. Based on our results, we conclude that NRG2 extracellular domain is shed by ADAM10 and BACE2 and the remaining fragment is further processed by γ-secretase.

## Materials and Methods

### Cell Culture

B16F10 (murine melanoma), MC38CEA (murine colon cancer cells expressing human carcinoembryonic antigen) [23], MEF (murine embryonal fibroblasts), MEF ADAM10^−/−^ (a gift from Prof. Paul Saftig, Christian-Albrechts University Kiel, Germany), and ADAM17^ΔZn/ΔZn^ MEF (a gift from Prof. Roy Black, at that time Amgen, Thousand Oaks, CA) were cultured at standard conditions in DMEM (BioWest) supplemented with 10% heat-inactivated, gamma-irradiated fetal bovine serum (BioWest). All cell cultures were screened for *Mycoplasma* contamination using PCR with *Mycoplasma* rDNA-specific probes.

### Construction of Expression Vectors

Total RNA was isolated from the brain of three-week old C57BL/6 mouse using Chomczyński and Sacchi method [24]. The tissue was obtained from the animal house at the Faculty of Biochemistry, Biophysics and Biotechnology in Kraków. Poly(A)^+^ fraction was obtained by incubating isolated RNA with oligo(dT)-cellulose tablets (Invitrogen) as described in [25]. Five hundred nanograms of poly(A)^+^ RNA was reverse-transcribed using ImProm II Reverse Transcriptase system (Promega) following manufacturer’s recommendations, using oligo(dT) primer. Coding sequences of NRG1 types I and III and NRG2 were PCR-amplified using HiFi HotStart DNA Polymerase (KAPA Biosystems) with primers listed in Supplementary Table 1. PCR products were resolved in 1% agarose gel in TAE buffer. Bands corresponding to sequences coding for NRG1 type I, NRG1 type III, or NRG2 were cut out, purified using Gel/PCR ME Mini Kit (Syngen Biotech) and cloned into pJET1.2/blunt (Thermo Scientific). The sequences of all constructs were confirmed with Sanger sequencing (all sequencing procedures were performed in Genomed S.A., Warsaw, Poland). During the cloning of *Nrg2*, four plasmids were sequenced. HA-tag and/or FLAG-tag were introduced to pJET1.2-NRG vectors using QuikChange technique as described in [26] or by inverse PCR using primers listed in Supplementary Table 1. FLAG- and/or HA-tagged NRGs were then subcloned into pLVX-IRES-puro plasmid (Clontech) using *Xho*I/*Xba*I restriction sites. The sequences coding for ADAM10, BACE1, and BACE2 were PCR-amplified from reverse-transcribed total RNA isolated from C57BL/6 mouse tissues with primers containing *Bam*HI, *Eco*RI, or *Not*I restriction sites (listed in Supplementary Table 1) and cloned into pJET1.2/blunt, then subcloned into LeGO-iT2 plasmid [27] (a gift form from Boris Fehse; Addgene plasmid # 27343), using *Bam*HI/*Not*I or *Bam*HI/*Eco*RI restriction sites. In the case of ADAM10, every resulting plasmid contained a frameshifting mutation in ADAM10 coding sequence; to restore the correct reading frame, a clone containing a single nucleotide deletion was subjected to QuikChange site-directed mutagenesis with primers that restore the correct reading frame. For generation of Sleeping Beauty transposon vector, a sequence coding for FLAG- and HA-tagged NRG2 was PCR-amplified with Phanta Max polymerase (Vazyme Biotech), digested with *Sfi*I and cloned into *Sfi*I-digested pSBbi-Pur [28] (a gift from Eric Kowarz; Addgene plasmid # 60523). The resulting vector was called pSBbi-Pur-NRG2ΔC, because it coded for NRG2 lacking a part of its predominant C-terminus.

The full-length C-terminal fragment of NRG2 was codon-optimized to decrease GC content, synthesized as double-stranded DNA (GeneArt Strings, Thermo Scientific), and cloned into PCR-amplified pSBbi-Pur-NRG2ΔC using NEBuilder HiFi DNA Assembly Mix (NEB). For generation of lentiviral vector encoding NRG2 containing the full-length C-terminal fragment, pSBbi-Pur-NRG2 was propagated in *dam*^−^*/dcm*^−^*E. coli* (NEB); a *Sal*I-*Bcl*I restriction fragment from pSBbi-Pur-NRG2 was ligated into the *Sal*I/*Bam*HI digested pLVX-IRES-puro-NRG2ΔC.

For generation of a lentiviral vector encoding full-length NRG2 under neuron-specific human synapsin I promoter (hSyn), NRG2 coding sequence was PCR-amplified from pSBbi-Pur-NRG2 and hSyn promoter was PCR-amplified from human genomic DNA. NRG2 and hSyn promoter sequences were inserted into *Xba*I/*Eco*RI-digested LeGO-G/BSD [27] (a gift from Boris Fehse, Addgene plasmid # 27354) using NEBuilder HiFi DNA Assembly Mix (NEB).

### Lentivirus Production and Generation of Stable Cell Lines by Transduction or Transfection

Lentiviral particles were produced in HEK293T cells as described previously [29]. Briefly, 293T cells were cotransfected with pLVX-IRES-puro or LeGO expression constructs, packaging plasmid psPAX2, and envelope plasmid pMD2.G (psPAX2 and pMD2.G, Addgene plasmids #12260 and #12259, respectively, gifts from Didier Trono) using Polyethylenimine HCl MAX, Linear, MW 40,000 (PEI; PolySciences) at a ratio of DNA to PEI 1:3. Pseudoviral particles were concentrated by centrifugation for 3 h at 23,000*g* at 4 °C and resuspended in serum-free DMEM. pLVX-IRES-puro-based vectors were titrated using QuickTiter Lentivirus Titer Kit (Lentivirus-Associated HIV p24; Cell Biolabs); LeGO-iT2-based vector titers were assessed by transduction of target cells with serial dilutions of concentrated media and estimation of tdTomato-positive fraction using flow cytometer (FACSCalibur, BD Bioscience). The cells were transduced in 12-well plates in the presence of 8 μg/ml polybrene at MOI 5. For pLVX-IRES-puro vectors, puromycin was added 48 h after transduction at a final concentration of 10 μg/ml for MEF, 5 μg/ml for MC38CEA, or 1.25 μg/ml for B16F10. MEF WT, ADAM10^−/−^, and ADAM17^ΔZn/ΔZn^ were seeded in 12-well plates. On the following day, the cells were transfected with 950 ng of pSBbi-NRG vector together with 50 ng of transposase-encoding vector pCMV(CAT)T7-SB100 [30] (a gift from Zsuzsanna Izsvak; Addgene plasmid # 34879) using jetPRIME reagent (Polyplus Transfection) according to the manufacturer’s protocol. One day after transfection, puromycin was added to the cell culture medium at a final concentration of 10 μg/ml.

### Treatment with Inhibitors

Inhibitors were dissolved in DMSO and used at the indicated final concentrations: BB-94 (Batimastat; Sigma-Aldrich) 10 μM; BB-2516 (Marimastat; Sigma-Aldrich) 10 μM; GM6001 (Tocris Bioscience) 25 μM; GI254023X (Sigma-Aldrich) 10 μM; PF-06649283 (Sigma-Aldrich) 10 μM; AZD3839 (Selleck Chemicals) 10 μM; DAPT (Sigma-Aldrich) 5 μM; deshydroxy-LY411575 (Sigma-Aldrich) 10 μM. The cells were cultured overnight in the presence of inhibitors or vehicle control (DMSO). The final concentration of DMSO in a culture medium did not exceed 0.1%.

### siRNA Transfection

Cells were plated in 12-well plates. On the following day, the cells were transfected with siRNA at a final concentration of 5 nM using Lipofectamine RNAiMAX (Thermo Scientific) according to manufacturer recommendations. RNA was isolated 48 h after transfection; protein was isolated 72 h after transfection. Trilencer-27 siRNA duplexes were purchased from OriGene. The silencing efficiencies of siRNA pool were evaluated using RT-qPCR and were in the range of 70–95%.

### Western Blotting

The cells were lysed in ice-cold RIPA buffer (50 mM Tris-HCl pH 7.4, 150 mM NaCl, 1% NP-40, 0.5% sodium deoxycholate, 0.1% SDS), enriched with 0.5% CHAPS, 5 mM EDTA and Halt Protease Inhibitor Cocktail (Thermo Scientific). Equal amounts of protein (in a range of 10–25 μg in individual experiments) were resolved on 8% polyacrylamide gels in Laemmli buffer. Cultured primary neurons were lysed directly in SDS-PAGE loading buffer. Collected media were centrifuged (5 min, 2000*g*) to remove cell debris and concentrated by centrifugation using Amicon Ultra 0.5 ml centrifugal units 10 MWCO (Merck) or by precipitation with ethanol. Concentrated media were subjected to SDS-PAGE and proteins were transferred onto PVDF membranes (Immobilon P, Merck). The membranes were stained with Ponceau-S to ensure equal protein loading, destained with TBS with Tween-20 (TBST), blocked with 5% skimmed milk in TBST and probed with following antibodies: rabbit anti-HA-tag (Abcam #9110, 1:10,000), rabbit anti-FLAG-tag (Proteintech #20543-1-AP, 1:1000), rabbit anti-ADAM10 (Abcam #1997, 1:5000), rabbit anti-ADAM17 (Thermo Scientific #PA5-17080, 1:2500), mouse anti-BACE1 (Santa Cruz Biotechnology, clone 61-3e7, 1 μg/ml), rabbit anti-BACE2 (Thermo Scientific #PA1-753, 1:1000), rabbit anti-β-actin (clone 13E5, Cell Signaling Technology, 1:5000), mouse anti-β-III-tubulin (Thermo Scientific, clone 2G10, 1 μg/ml) and corresponding HRP-conjugated secondary antibodies: goat anti-rabbit Ig (Sigma-Aldrich, 1:20,000) or goat anti-mouse IgG (BD Pharmingen, 1:20,000). Bands were developed with Immobilon Western Chemiluminescent HRP Substrate (Merck) and visualized with a luminescence imaging platform Fusion FX (Vilber Lourmat). The exposition time was set to “auto.” Band intensities were quantified using Fiji software [31]; chemiluminescent signal was normalized to the total protein amount in each lane visualized by Ponceau staining (unless stated otherwise). In the case of primary neurons, after NRG2 signal was developed, membranes were subjected to antibody removal in the mild stripping buffer (200 mM glycine, pH 2.2, 0.1% SDS, 1% Tween-20), blocking and reprobing with anti-β-III-tubulin. NRG2 signal was normalized to the signal of neuron-specific marker, β-III-tubulin. The color of images was inversed using Fiji software. If needed, contrast of images was adjusted and the change has been marked in the picture and its description. Original, unprocessed images and chemiluminescent images merged with white light images of the membranes with PageRuler Prestained Protein Ladder, 10 to 180 kDa (Thermo Scientific) are included in the Electronic Supplementary Material together with pictures of membranes stained with Ponceau-S (if available). The images were merged in Fusion FX software. The molecular mass of NRG2 was estimated by measuring the relative migration distance of the protein and the standards of known molecular masses, plotting the distances of the standard proteins versus their molecular masses on a semi-log graph, fitting a linear curve and calculating the molecular mass of NRG2 based on the equation of the standard curve.

### Protein Dephosphorylation and Deglycosylation

Protein lysates were dephosphorylated using FastAP Thermosensitive Alkaline Phosphatase (Thermo Scientific) as recommended by the manufacturer. Briefly, 20 μg of proteins from the lysates of cells treated with DAPT was incubated with 10 U of FastAP in 1 × FastAP reaction buffer at 37 °C for 1 h. Proteins form the cell lysates or concentrated media were deglycosylated using recombinant PNGase F (Promega) according to manufacturer’s recommendations. Dephosphorylated or deglycosylated proteins were subjected to SDS-PAGE and Western blotting analysis performed with antibodies anti-FLAG or anti-HA as described above.

### RNA Isolation, Reverse Transcription, and RT-qPCR

Total RNA was isolated by the modified guanidinium isothiocyanate and phenol/chloroform extraction using Fenozol reagent (A&A Biotechnology), followed by DNAse I digestion and purification using Clean-Up RNA Concentrator (A&A Biotechnology) as recommended by the manufacturer. Equal amounts of RNA (1 μg) were reverse-transcribed using M-MLV transcriptase (Promega) and oligo(dT)_15_ primer according to manufacturer’s recommendations. RT-qPCR was performed using AceQ qPCR SYBR Green Mix (Vazyme Biotech) on Eco Real-Time PCR System (Illumina). Reaction for each data point was performed in duplicates. RNA expression was normalized to a geometric mean of at least two of the following reference genes: *eEF2*, *Tbp*, *PolR2b*, *ActB*. Primes used for qPCR experiments are listed in Supplementary Table 2.

### Immunohistochemistry

Brains of C57BL/6J healthy, adult mice (6–8 weeks old) were obtained from the Animal Facility of the Faculty of Biochemistry, Biophysics and Biotechnology, Jagiellonian University, Kraków, Poland, pursuant to Directive 2010/63/EU of the European Parliament and of the Council. The brains were fixed in 4% paraformaldehyde in PBS, embedded in paraffin, sectioned coronally at a thickness of 7 μm using a microtome, and mounted onto Superfrost Plus slides (Thermo Scientific). The slides were heated for 1 h at 60 °C in an oven, then deparaffinized with a series of xylene and graded ethanol washes. Endogenous peroxidase activity was blocked by incubating the sections in 0.3% H_2_O_2_ in 40% methanol in PBS overnight. After brief TBS washes, the sections underwent antigen retrieval in 10 mM citrate buffer, 0.05% Tween-20, pH 6.0, in a wet autoclave at 121 °C for 5 min. After three washes in TBST, the sections were incubated in blocking solution (20% normal goat serum, 3% BSA in TBS) for 30 min, and then for additional 30 min in blocking solution containing Mouse BD Fc Block (rat anti-mouse CD16/CD32, clone 2.4G2, BD Biosciences). Endogenous mouse immunoglobulins were blocked with unstained Fab fragments of goat anti-mouse IgG (Jackson ImmunoResearch, #115-007-003) in TBS at 100 μg/ml for at least 2 h at room temperature. After three washes in TBST, the sections were incubated overnight at 4 °C with mouse anti-NRG2 (Sigma-Aldrich, clone 8D11, 2 μg/ml) or isotype control (mouse anti-human CEA, clone COL-1, Zymed), diluted in 10% normal goat serum, 1% BSA in TBST (dilution buffer). After washing six times for 5 min in TBST, the slices were incubated with SuperBoost Goat anti-Mouse Poly HRP (Thermo Scientific) for 1 h at room temperature, washed six times with TBST and then subjected to signal amplification using Alexa Fluor 488 Tyramide (Thermo Scientific) for 10 min at room temperature. Following five washes in TBST, the sections were subjected to microwave treatment in 10 mM citrate buffer, 0.05% Tween-20, pH 6.0, (2 min in boiling solution), to inactivate HRP. After incubation in blocking solution containing Mouse Fc Block, the slices were subjected to staining using rabbit anti-BACE2 antibody (Thermo Scientific #PA1-753, 1:1000) or rabbit IgG isotype control (Novus Biologicals, #NB810-56910), employing the same staining procedure as described for NRG2 staining but using SuperBoost Goat anti-Rabbit Poly HRP and Alexa Fluor 546 Tyramide (Thermo Scientific). In some sections, anti-BACE2 and anti-rabbit IgG were substituted with dilution buffer and then incubated with Alexa Fluor 546 Tyramide, to ensure that HRP from the first round of staining was successfully inactivated. Finally, after additional microwave treatment in boiling citrate buffer for 8 min in order to remove mouse anti-NRG primary antibodies, TBST washes, and blocking, the sections were incubated with mouse anti-β-III-tubulin (Thermo Scientific, clone 2G10, 4 μg/ml) or isotype control antibodies (mouse IgG2a Isotype Control, Sigma-Aldrich, clone UPC-10) followed by Alexa Fluor 647-conjugated F(ab′)_2_ of goat anti-mouse IgG (Jackson ImmunoResearch #115-606-062, 1:200). After dipping in 70% ethanol, the sections were transferred to a filtered solution of 0.3% Sudan Black B (Sigma-Aldrich) in 70% ethanol for 30 min, followed by two brief washes in 70% ethanol and PBS. The sections were mounted with Vectashield Antifade Mounting Medium with DAPI (Vector Laboratories) and coverslipped. Images were taken on Leica DM6 B microscope equipped with Leica DMC5400 camera. Images were adjusted for overall brightness and contrast in Fiji software.

### Hippocampal Neuron Culture

Primary hippocampal neurons were isolated from the brains of adult C57BL6/J mice at 4–8 weeks of age according to a published protocol [32] with minor modifications. Briefly, isolated hippocampi were minced with a sterile razor and digested for 30 min at 30 °C in papain (2 mg/ml; Worthington Biochemical Corporation) in digestion buffer (68.9 mM NaCl, 5.3 mM KCl, 0.81 mM MgCl_2_, 0.88 mM NaHCO_3_, 0.91 mM NaH_2_PO_4_, 10 mM HEPES, 25 mM D-glucose, 0.23 mM sodium pyruvate, 10 mM MOPS, pH 7.3). Then DNase I (200 U/ml; Sigma-Aldrich) and MgCl_2_ (to a final concentration of 5 mM) were added and the tissue was incubated for additional 5 min. Papain/DNase solution was replaced with Hibernate-A/B27 Plus medium (Thermo Scientific) and the tissue was titurated with fire-polished, siliconized Pasteur pipettes with descending diameters of openings and fractionated on a four step Optiprep (Sigma-Aldrich) gradient. The neuronal fraction was collected, rinsed with Hibernate-A/B27 Plus and subjected to additional debris removal step, in which the cells were mixed with Optiprep (9% final concentration of iodixanol) in Hibernate-A/B27 Plus, overlayed with Hibernate-A/B27, and centrifuged at 3000*g* for 10 min. The top layers were discarded and the layer containing neurons was washed with Hibernate-A/B27 Plus. The cell pellet was resuspended in Neurobasal-A/B27 Plus medium (Thermo Scientific) containing 0.5 mM Ultraglutamine (Lonza), gentamicin (5 μg/ml), and mouse FGF2 (10 ng/ml; BioLegend). Neurons were plated on poly-d-lysine-coated coverslips. One fourth of the medium was replaced twice a week. The cells were transduced with lentiviral vectors encoding NRG2 at DIV4. All experiments were performed on cells at ≥DIV14.

## Results

### Obtaining a Full-Length Murine NRG2 Coding Sequence

To investigate proteolytic processing of murine NRG2, we decided to overexpress NRG2 in three different cell lines. NRG2 expression was reported to be the highest in specific regions of the brain and therefore we decided to amplify NRG2 coding sequence from RNA isolated from a mouse brain tissue. *Nrg2* gene contains 12 exons; therefore, it may produce a large number of alternatively spliced variants. The full-length NRG2 sequence that we have amplified resembles the predicted transcript variant X1 (XM_006525461.3) except it lacks a fragment located in the last exon. The resulting NRG2 protein is denoted as NRG2ΔC and the sequence of its open reading frame is included in the Electronic Supplementary Material.

### ADAM10 and BACE2 Are the Key Enzymes Involved in NRG2 Ectodomain Shedding

**Fig. 1:**
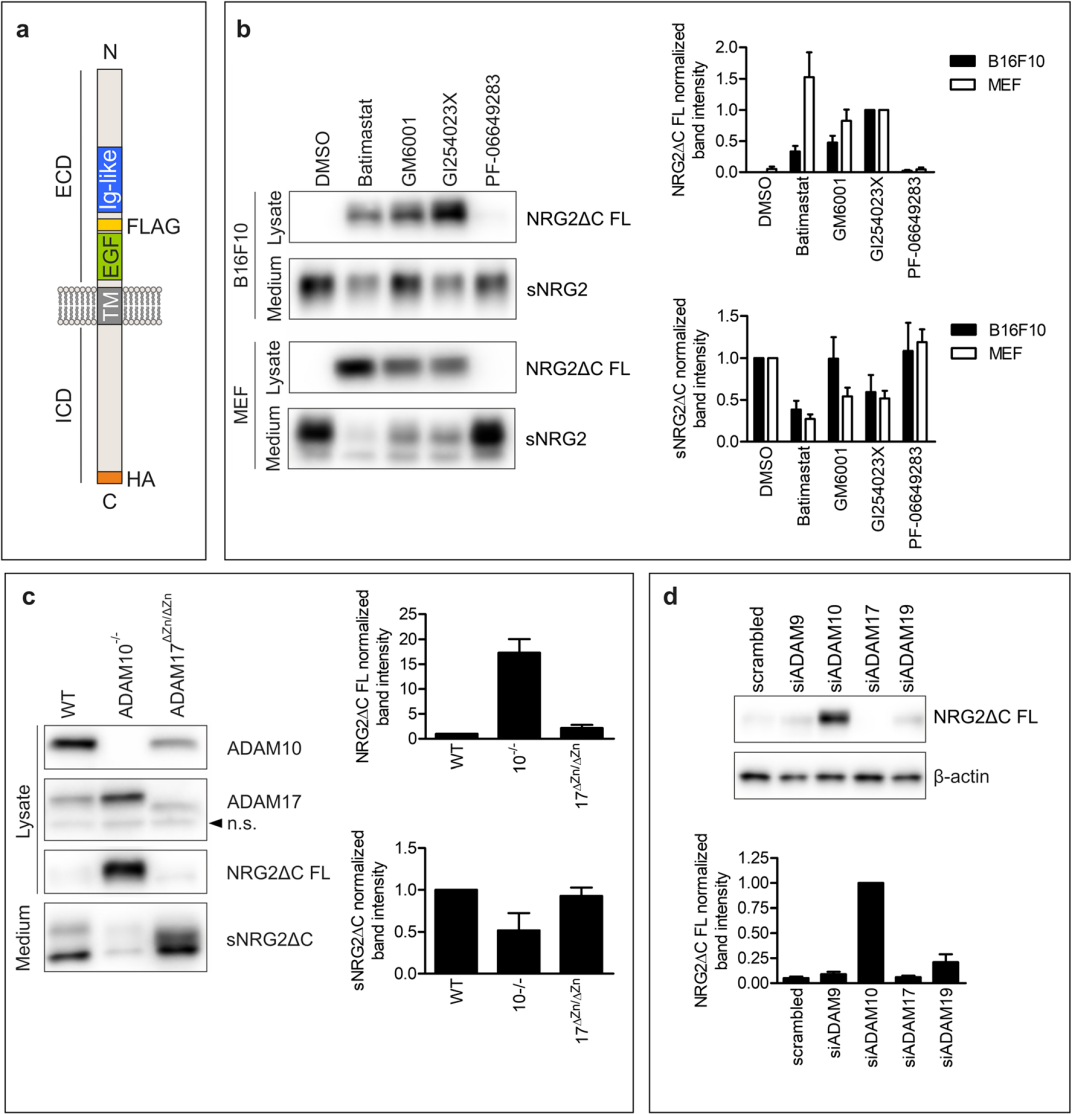
Mouse NRG2 ectodomain is shed by an enzyme belonging to metalloproteases. **a** Schematic representation of NRG2 used in this study. A FLAG-tag was inserted in the extracellular domain of NRG2 (ECD) between Ig-like and EGF-like domains. HA-tag was inserted immediately after NRG2 coding sequence at the C-terminus. **b** Western blotting analysis of the levels of soluble NRG2ΔC in the culture medium (sNRG2ΔC, detected with anti-FLAG antibody) and full-length NRG2ΔC (NRG2ΔC FL, detected with anti-HA antibody) in the lysates of B16F10 and MEF cells incubated with metalloprotease inhibitors or general BACE inhibitor. Right panel: quantification of WB signals. Graph represents fold changes of NRG2ΔC-linked chemiluminescent signals. For analysis of NRG2ΔC FL, the chemiluminescent signal of the sample from the cells treated with GI254023X was set as one and for analysis of sNRG2ΔC the chemiluminescent signal of the sample from the cells treated with vehicle (DMSO) was set as one. Quantification of band intensities from four (B16F10) or three (MEF) independent experiments is shown as mean value (MV) ± standard error of the mean (SEM). **c** Western blotting analysis of NRG2ΔC shedding in wild-type (WT) murine embryonal fibroblasts (WT MEF), ADAM10-deficient (ADAM10^−/−^ or 10^−/−^) MEF and MEF that lack ADAM17 catalytic activity (ADAM17^ΔZn/ΔZn^ or 17^ΔZn/ΔZn^). Band intensities in WT MEF cells were set as one. Mean values ± SEM from three independent experiments are shown. **d** Western blotting analysis of full-length NRG2ΔC in lysates of B16F10 cells transduced with a vector coding for C-terminally HA-tagged NRG2ΔC upon silencing of ADAM9, ADAM10, ADAM17, or ADAM19 with siRNA pools. Graph represents the fold change of band intensities normalized to β-actin; band intensity of a sample from the cells transfected with ADAM10 siRNA is set as one. Mean values ± SEM from four independent experiments are shown

To study NRG2 processing in cells, we have chosen B16F10, MC38CEA, and MEF cells. All of these cell lines express NRG2 as well as its receptor and thus we expected them to produce NRG2-competent sheddase(s). We transduced the cells with vectors coding for NRG2ΔC with introduced common tags to enable simple detection with commercially available antibodies. The cells stably expressed either single-tagged NRG2ΔC (C-terminal HA-tag) or double-tagged NRG2ΔC (FLAG-tag within ectodomain and C-terminal HA-tag; Fig. 1a). In all cell lines, NRG2ΔC was efficiently processed because the full-length form of NRG2ΔC was barely detectable despite a high level of transgene mRNA expression (data not shown). We first inhibited activity of metalloproteases and BACE in B16F10 and MEF cells with small-molecule inhibitors. Metalloprotease- and BACE inhibitors were chosen by analogy with NRG1, which was shown to be processed by several ADAM metalloproteases and BACE1. In both cell lines tested, inhibition of metalloproteases activity by Batimastat, GM6001, and GI254023X resulted in an increase in the level of full-length NRG2ΔC in cell lysates (detected with antibodies recognizing HA epitope; Fig. 1b). This was accompanied by a strong reduction of the level of the soluble NRG2 ectodomain (detected with anti-FLAG antibodies) in conditioned media (Fig. 1b). We did not detect any substantial changes in NRG2ΔC levels upon inhibition of BACE with PF-06649283. GI254023X is a potent and selective ADAM10 inhibitor (with high selectivity over ADAM17 [33]); therefore, ADAM10 is most likely the main protease responsible for NRG2ΔC shedding in the tested cell lines. Moreover, in ADAM10^−/−^ fibroblasts, the level of full-length NRG2ΔC was strongly elevated and the level of soluble NRG2ΔC was strongly reduced in comparison with wild-type MEF cells, or cells that lack ADAM17 protease activity (ADAM17^ΔZn/ΔZn^ MEF; Fig. 1c). It is in agreement with the results of experiments utilizing siRNA to silence the expression of ADAM9, ADAM10, ADAM17, or ADAM19—only siRNA against ADAM10 was able to inhibit shedding of NRG2ΔC (Fig. 1d and Supplementary Fig. 1a). Although we observed an elevated level of full-length NRG2ΔC in cells, in which ADAM19 was silenced with one of three tested siRNAs, but this resulted most probably from the increased total level of NRG2ΔC, because these cells showed augmented *Nrg2* mRNA expression (Supplementary Fig. 1b).

**Fig. 2:**
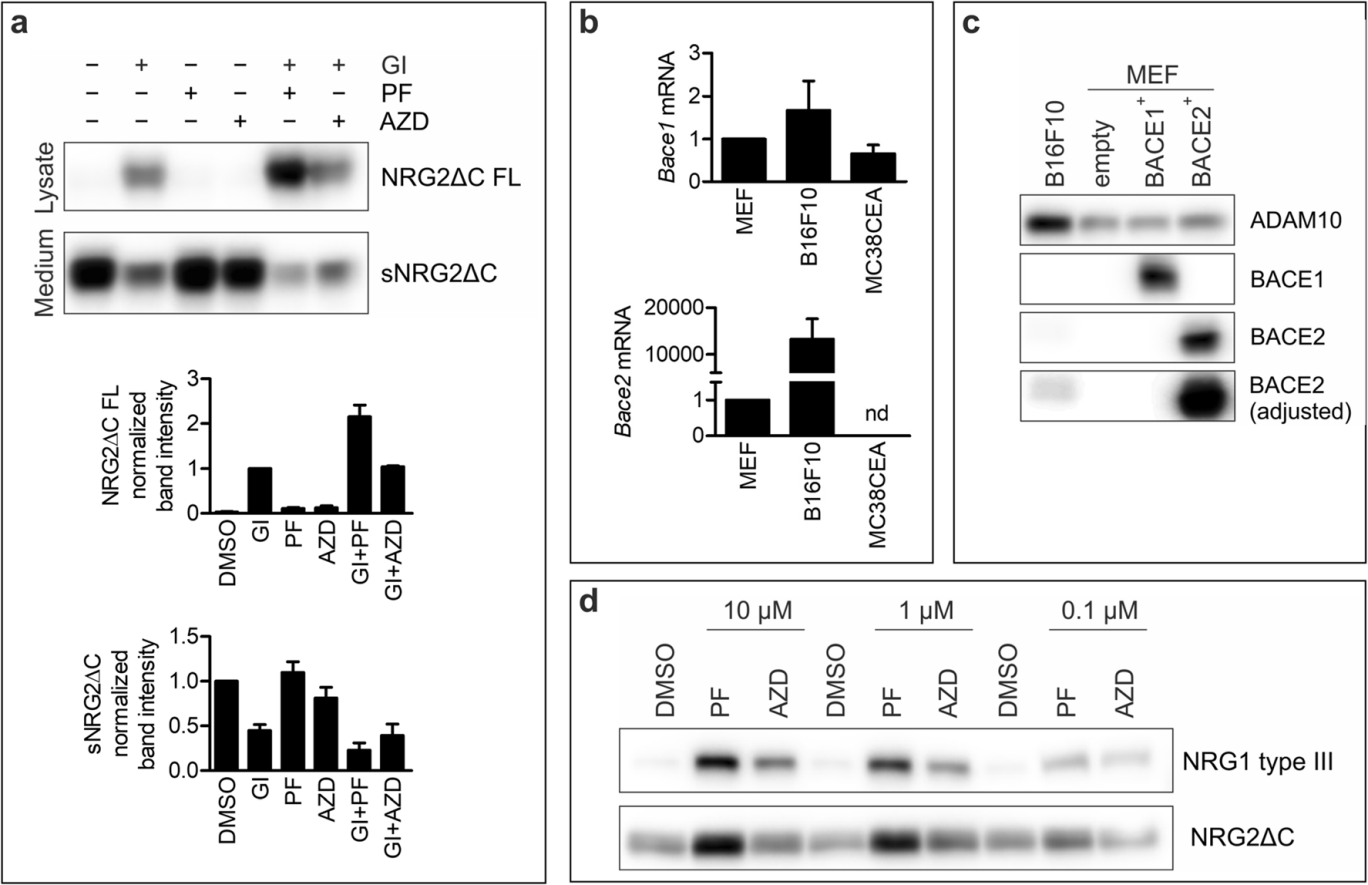
Inhibitors of ADAMs and BACEs affect NRG2 shedding. **a** Western blotting analysis of soluble NRG2ΔC (sNRG2ΔC) in the culture medium and full-length NRG2ΔC (NRG2ΔC FL) in the lysates of B16F10 cells treated with ADAM10-specific inhibitor GI254023X (GI), general BACE inhibitor PF-06649283 (PF), BACE1-specific inhibitor AZD3839 (AZD), or combinations of two inhibitors. Lower panel: quantification of WB signals. Graph represents mean values of fold changes of NRG2ΔC-linked chemiluminescent signals ± SEM of three independent experiments. For analysis of NRG2ΔC FL, the chemiluminescent signal of the sample from the cells treated with GI254023X was set as one and for analysis of sNRG2ΔC the chemiluminescent signal of the sample from the cells treated with vehicle (DMSO) was set as one. **b** RT-qPCR analysis of *Bace1* and *Bace2* mRNA levels in MEF, B16F10 and MC38CEA cells. Data is shown as MV ± SEM from three independent experiments. **c** Western blotting analysis of ADAM10, BACE1 and BACE2 protein levels in B16F10- and MEF cells transduced with an empty vector or BACE1-, or BACE2-encoding vectors. The PVDF membrane incubated with anti-BACE2 antibody was cut for bands visualization because of a strong, unspecific signal detected below BACE2-specific one. The original image from the uncut membrane is available in Electronic Supplementary Material. The contrast of BACE2 image was adjusted to enable visualization of BACE2 expression in B16F10 cell lysate. Representative images from three independent experiments are shown. **d** Western blotting analysis of NRG1 type III or NRG2ΔC shedding in B16F10 cells transduced with vectors coding for HA-tagged NRG1 type III or NRG2ΔC in the presence of vehicle (DMSO) or different concentrations of general BACE inhibitor PF-06649283 or BACE1-specific inhibitor AZD3839. All cells were treated simultaneously with Batimastat to reduce ADAM10- and/or ADAM17-mediated NRGs shedding. Representative images from three independent experiments are shown

However, inhibition of metalloprotease activity in B16F10 did not prevent NRG shedding and significant amounts of soluble NRG2ΔC were still detected in cell culture medium. To further investigate if proteases other than ADAM10 are involved in the shedding of NRG2ΔC ectodomain, we incubated the cells simultaneously with ADAM10 inhibitor GI254023X and either general BACE inhibitor PF-06649283 or selective BACE1 inhibitor AZD3839. We observed that in B16F10, the combination of ADAM10 inhibitor with BACE inhibitor PF-06649283 further diminished the level of soluble NRG2ΔC compared to that in the cells treated with GI254023X only, and this was accompanied by accumulation of full-length, unprocessed NRG2ΔC (Fig. 2a). This effect was not observed in MEF and MC38CEA cells (data not shown). The levels of both soluble NRG2ΔC (sNRG2ΔC) and full-length NRG2ΔC in the samples from B16F10 treated simultaneously with GI254023X and BACE1 selective inhibitor AZD3839 were comparable to those in the cells incubated with ADAM10 inhibitor only. RT-qPCR demonstrated that all cell lines expressed *Bace1* mRNA; significant amounts of *Bace2* transcript were present only in B16F10 cells (Fig. 2b). Moreover, among these unmodified lines, BACE2 protein is detectable only in B16F10 cells (Fig. 2c, bottom panel). Therefore, we concluded that besides ADAM10, also BACE2 is capable of NRG2 shedding. Using B16F10 cells that overexpressed HA-tagged NRG1 type III (which may be processed by both ADAM10 and ADAM17 as well as BACE1) or NRG2ΔC, we confirmed that the concentration of AZD3839 used in this study was sufficient to inhibit BACE1-mediated cleavage (Fig. 2d). Moreover, we showed that AZD3839 was specific towards BACE1, because it did not inhibit processing of NRG2ΔC by BACE2, in contrast to PF-06649283, which inhibited both BACE1 and BACE2 activity towards NRG1 and NRG2 shedding, respectively.

**Fig. 3:**
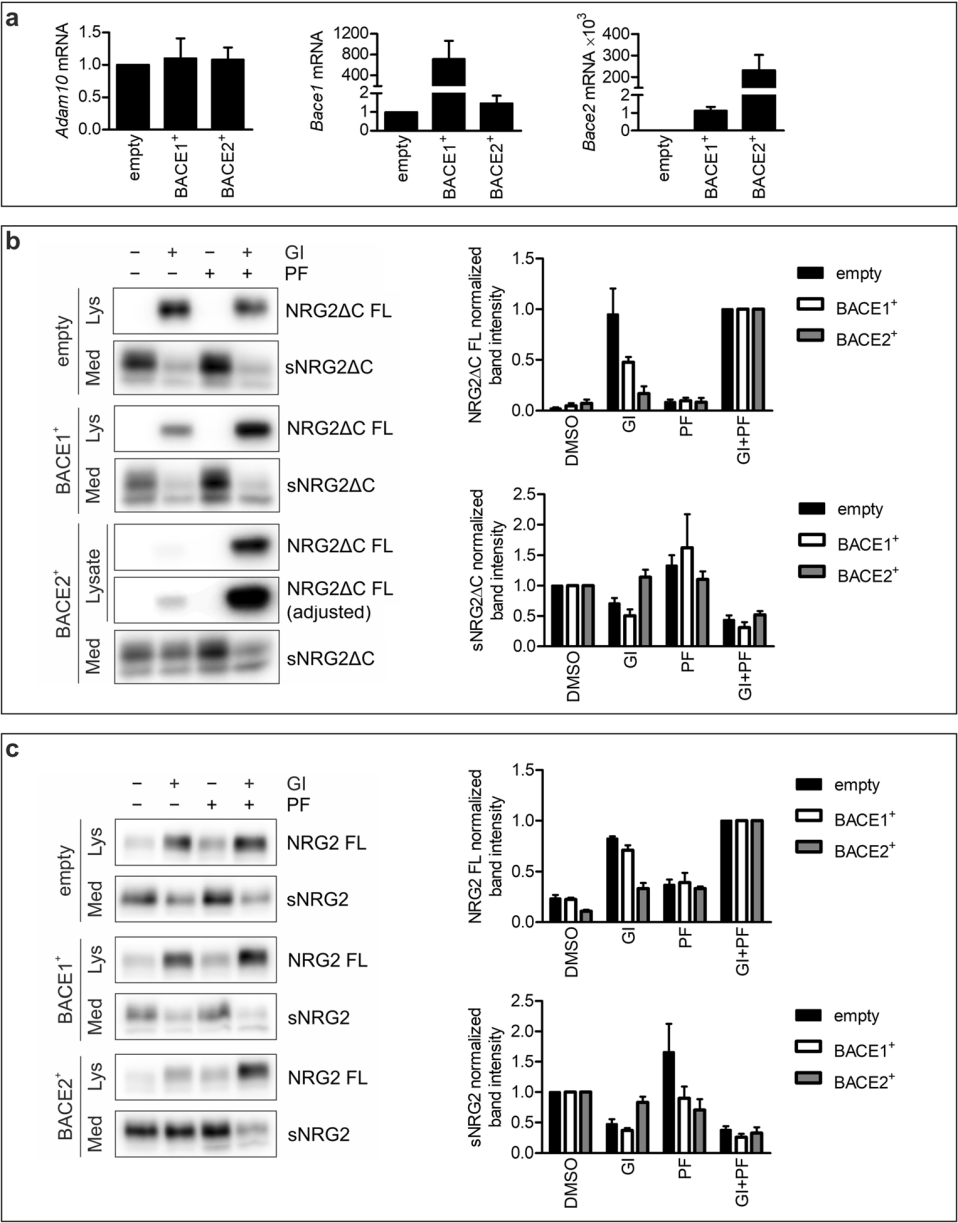
ADAM10 and BACE2 are responsible for generation of soluble NRG2. **a** RT-qPCR analysis of the levels of *Adam10*, *Bace1* and *Bace2* transcripts in MEF cells transduced with an empty vector or vectors coding for murine BACE1 or BACE2. Data is shown as MV ± SEM from three independent experiments. **b**, **c** Western blotting analysis of NRG2ΔC (**b**) or NRG2 (**c**) shedding in the presence of ADAM10-specific inhibitor GI254023X (GI), general BACE inhibitor PF-06649283 (PF), or their combination in MEF cells transduced with an empty vector or vectors coding for murine BACE1 or BACE2. The contrast of NRG2ΔC FL image was adjusted to enable visualization of NRG2ΔC FL in BACE2^+^ MEF cells. Right panels: Quantification of WB signals. Graph represents the MV of fold change of band intensities ± SEM from five independent experiments. For analysis of NRG FL, the chemiluminescent signal of the sample from the cells treated with GI254023X + PF-06649283 (GI + PF) was set as one and for analysis of sNRG—the chemiluminescent signal of the sample from the cells treated with vehicle (DMSO)

To further confirm the contribution of BACE2, and not BACE1, in NRG2ΔC proteolysis, we overexpressed BACE1 and BACE2 in MEF cells, generating BACE1^+^ MEF and BACE2^+^ MEF cell lines (Figs. 2c and 3a). All cell lines expressed comparable levels of ADAM10 (Figs. 2c and 3a). In the cells transduced with an empty vector, the levels of full-length NRG2ΔC as well as sNRG2ΔC were comparable between cells incubated with ADAM10 inhibitor and cells treated with both ADAM10- and BACE inhibitors (Fig. 3b). This finding is in agreement with the results obtained for unmodified MEF cells. Overexpression of BACE1 did not abolish suppression of NRG2ΔC shedding by ADAM10 inhibitor, GI254023X; however, it led to increased sensitivity of NRG2ΔC shedding to general BACE inhibitor, PF-06649283. Although, this inhibitor alone did not lead to the increase in membrane content of NRG2ΔC, but it significantly potentiated the inhibitory effect of GI254023X (Fig. 3b). It may be due to the fact that in BACE1-overexpressing cells, the levels of *Bace2* mRNA were also elevated in comparison with the cells transduced with an empty vector (Fig. 3a, rightmost panel). In contrast to overexpression of BACE1, transduction of MEF cells with a vector encoding BACE2 led to a substantial reduction of the level of the full-length form of NRG2ΔC. Treatment of BACE2^+^ MEF cells with ADAM10 inhibitor GI254023X resulted in only slight increase in the level of full-length NRG2ΔC; despite similar amounts of protein loaded on the gel, the time required for optimal detection of chemiluminescent signal was much longer for membranes with lysates from BACE2^+^ cells than from control or BACE1^+^ cells (e.g., ~ 80 s vs ~ 20 s). Also, the decrease in the amount of sNRG2ΔC upon ADAM10 inhibition in BACE2^+^ MEF was barely detectable. A pronounced inhibition of NRG shedding was visible only after incubation of the cells with both GI254023X and PF-06649283 (Fig. 3b). Because soluble NRG2ΔC fragments generated upon metalloprotease or BACE inhibition migrated with the same apparent molecular mass, it suggested that ADAM10 cleavage site is located in close proximity to BACE2 cleavage site. To confirm that a canonical form of NRG2 containing a full C-terminal fragment (denoted as NRG2) undergoes proteolytic processing by the same enzymes as NRG2ΔC, we overexpressed BACE1 and BACE2 in MEF cells overexpressing NRG2. Similarly to NRG2ΔC, NRG2 was efficiently cleaved both by ADAM10 and BACE2, but not BACE1 (Fig. 3c). Moreover, NRG2 seems to be even more sensitive to BACE2 cleavage than NRG2ΔC, because the effect of BACE-specific inhibitor, PF-06649283 on inhibition of NRG2 shedding is comparable to the effect of ADAM10-specific inhibitor, GI234023X.

**Fig. 4:**
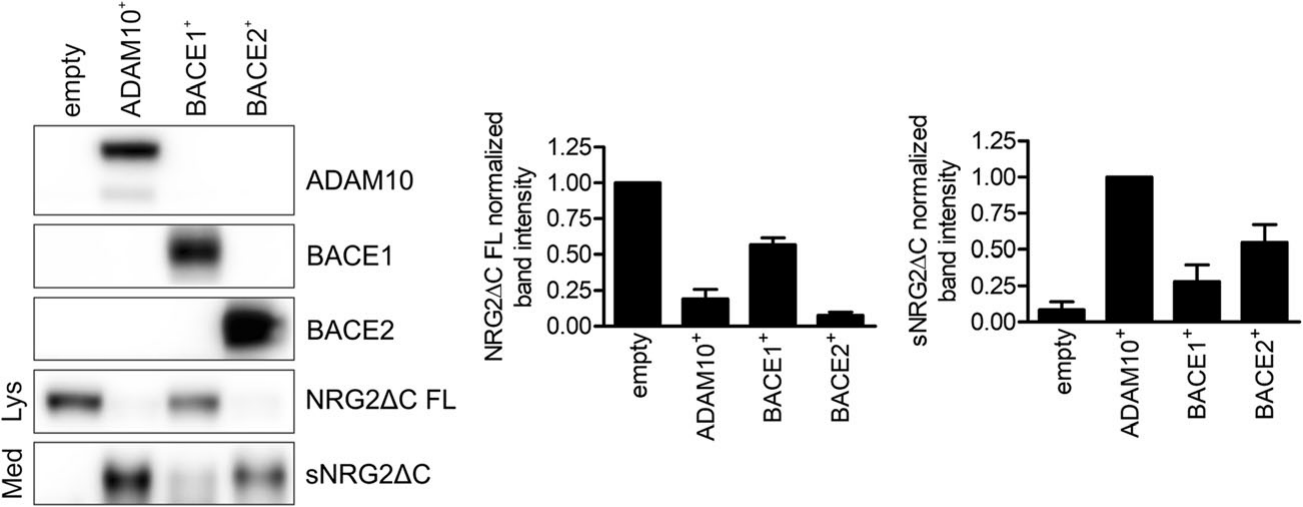
BACE2 may compensate for a lack of ADAM10 in NRG2 shedding. Western blotting analysis of ADAM10, BACE1, BACE2 and NRG2ΔC levels in ADAM10-deficient MEF cells transduced with an empty vector or a vector encoding murine ADAM10, BACE1, or BACE2. Lower panel: quantification of WB signals. Graph represents mean values of band intensities ± SEM from five (for ADAM10^+^- and BACE2^+^ cells) or three (for BACE1^+^ cells) independent experiments. Band intensities in samples from cells transduced with empty vector (for NRG2ΔC FL) or ADAM10-coding vector (for sNRG2ΔC) were set as one

In ADAM10-knockout MEF cells, we detected only a small amount of sNRG2ΔC in conditioned medium, while full-length NRG2ΔC was present in cell lysates in large quantities (Fig. 4). Restoration of ADAM10 expression in those cells resulted in enhanced liberation of soluble NRG2ΔC ectodomain to the cell culture medium. Increased sNRG2ΔC level was accompanied by strong decline in the full-length NRG2ΔC level in the cell lysates (Fig. 4). Overexpression of BACE1 was not able to compensate for a lack of ADAM10; full-length NRG2ΔC in lysates was present in a much larger quantity than in the lysates of cells overexpressing ADAM10, while the level of sNRG2ΔC was substantially lower than that observed for ADAM10^+^ cells. In contrast, overexpression of BACE2 in ADAM10-knockout MEF cells resulted in significantly diminished full-length NRG2ΔC- and increased sNRG2ΔC levels in the cell lysate and conditioned medium, respectively. Collectively, these results indicate that both ADAM10 and BACE2 are able to efficiently shed NRG2 ectodomain; BACE1 may also be involved in NRG2 proteolysis, although this process seems to be much less efficient than these catalyzed by ADAM10 or BACE2. The weak influence of BACE1 on NRG2ΔC processing (Figs. 3b and 4) was not observed in experiments performed with NRG2 (Fig. 3c), thus probably BACE1 has only marginal, if any, effect on the shedding of the dominant natural form of NRG2.

**Fig. 5:**
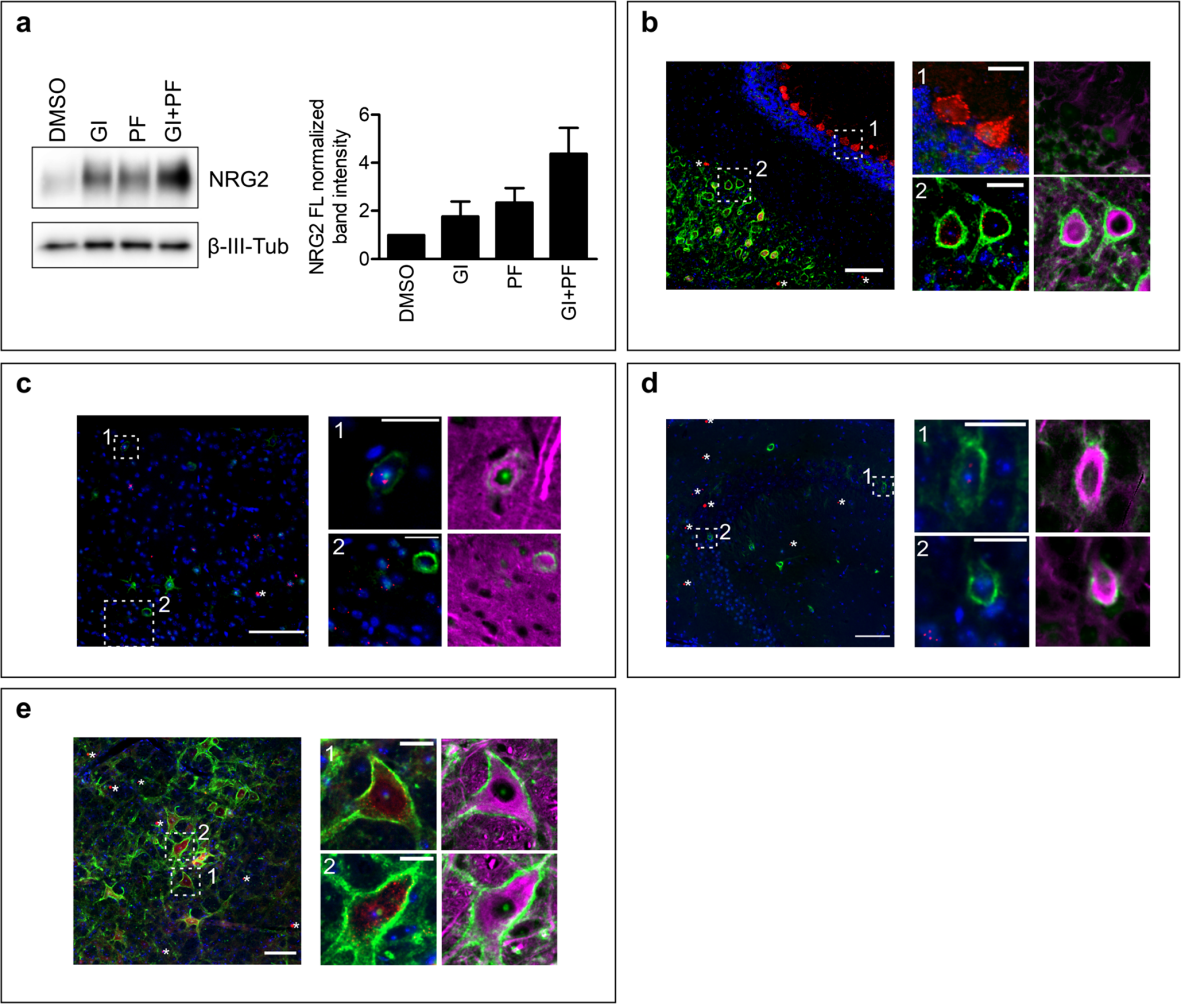
ADAM10 and BACE2 may serve as NRG2 sheddases in the nervous system. **a** Western blotting analysis of NRG2 shedding in the presence of ADAM10-specific inhibitor GI254023X (GI), general BACE inhibitor PF-06649283 (PF), or their combination in primary hippocampal neurons transduced with vectors coding for NRG2. Graph represents the fold change of band intensities normalized to β-III-tubulin (β-III-tub). Bands intensities of a sample from the cells treated with DMSO is set as one. Mean values ± SEM from three independent experiments are shown. **b**–**e** Immunohistochemical staining of cerebellum (**b**), cerebral cortex (**c**), CA3 region of the hippocampus (**d**) and medulla oblongata (**e**). NRG2 is shown in red, BACE2 in green, β-III-tubulin in magenta and nuclei (DAPI) in blue. Asterisks show artefactual signal, obtained presumably due to the use of anti-mouse IgG antibody on mouse tissue; the same staining pattern was observed in isotype control and secondary-only control. Scale bars: 100 μm and 25 μm for magnified areas. Representative images from five animals are shown

To confirm the relevance of identified NRG2 sheddases to neuronal cells, we isolated hippocampal cells, which supposedly express NRG2 as well as both ADAM10 and BACE2 and transduced them with a lentiviral vector coding for full-length NRG2 under neuron-specific human synapsin I promoter. The cells were subjected to ADAM10 and BACE inhibitors and the levels of unprocessed NRG2 were analyzed by western blotting (Fig. 5a). In agreement with results obtained for non-neuronal, BACE2-expressing cell lines, inhibition of either ADAM10 or BACEs resulted in moderate suppression of NRG2 shedding, while simultaneous treatment of neurons with ADAM10- and general BACE inhibitors led to the strong accumulation of unprocessed NRG2 in the cells. Assuming that BACE1 is not able to shed NRG2, the result supported the notion that both ADAM10 and BACE2 may play a role of NRG2 sheddase in neurons.

To further confirm the possible physiological significance of BACE2 in NRG2 shedding, we performed double immunocytochemistry on mouse brain sections with antibodies against BACE2 and NRG2, in search for the cells that show their co-expression. First, we tested the specificity of the antibodies on MEF cells transfected with vectors coding for mouse BACE1 or BACE2, or MEF ADAM10^−/−^ cells transfected with a plasmid encoding mouse NRG2 (Supplementary Fig. 2). Both antibodies are suitable for immunofluorescent staining; weak non-specific signal from anti-BACE2 antibody is observed in the nucleus, while the specific signal is found outside the nucleus. Anti-BACE2 antibody does not cross-react with BACE1 (Supplementary Fig. 2).

For immunohistochemistry, we used the most sensitive fluorescent imaging method for the detection of low abundance targets, which utilize tyramide amplification. In line with previous reports [4, 5, 34], the highest NRG2 immunoreactivity was found in cerebellar Purkinje cells; we also observed less abundant signals in other areas, including cerebral cortex, cerebellar granule cells, cerebellar deep nuclei, hippocampus, striatum, thalamus, midbrain, and medulla. Cerebellar Purkinje cells were not stained with anti-BACE2 antibody (Fig. 5b); BACE2-positive cells were observed in the cerebral cortex, but NRG2-positive cells were rarely found positive for BACE2 (Fig. 5c). Some neurons immunoreactive for NRG2 in the CA3 region of the hippocampus were also stained for BACE2; however, majority of the NRG2-positive cells were negative for BACE2 and vice versa (Fig. 5d). Hippocampal granule cells were positive for NRG2, but results obtained with anti-BACE2 antibody are inconclusive (data not shown). However, in the cerebellar deep nuclei and medulla, most of NRG2-immunoreactive cells were also positive for BACE2 (Fig. 5b, e). Both BACE2- and NRG2-positive cells were stained with anti-β-III-tubulin antibody (Fig. 5b–e), confirming their neuronal origin.

### NRG2 Is a Substrate for γ-Secretase

**Fig. 6:**
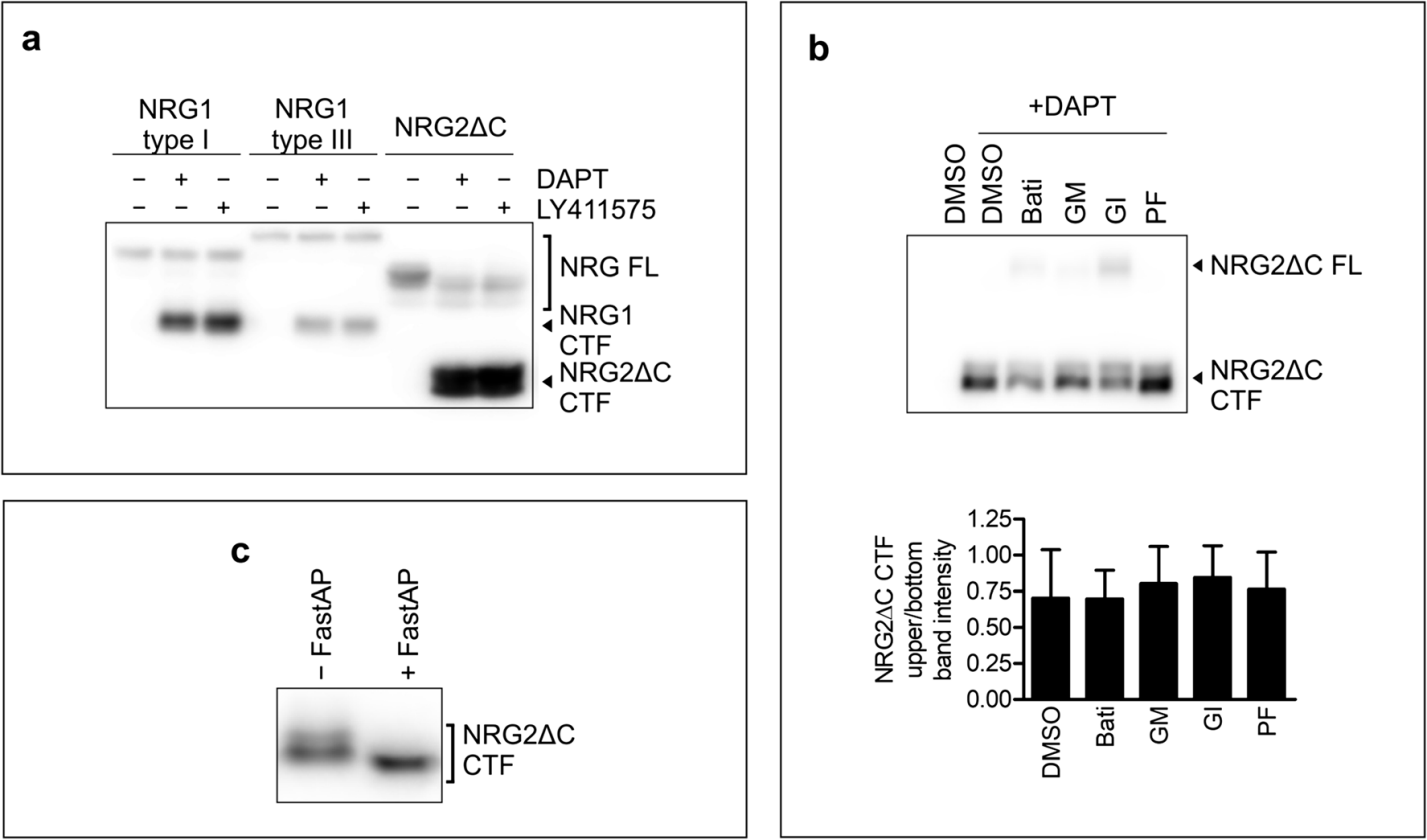
NRG2 is a substrate for γ-secretase. **a** Western blotting analysis of NRG2ΔC C-terminal fragment (CTF) accumulation in B16F10 cells transduced with C-terminally HA-tagged NRG2ΔC upon γ-secretase inhibition with DAPT or deshydroxy-LY411575 (denoted as LY411575). A representative image from three independent experiments is shown. **b** Western blotting analysis of NRG2ΔC C-terminal fragment accumulation in B16F10 cells transduced with C-terminally HA-tagged NRG2ΔC upon simultaneous inhibition of metalloprotease (with Batimastat, GM6001, or GI254023X) or BACE (with PF-06649283) and gamma-secretase inhibition with DAPT. A representative image from three independent experiments is shown. **c** In vitro dephosphorylation of NRG2ΔC CTF with alkaline phosphatase (FastAP). A representative image from three independent experiments is shown

In analogy to NRG1 processing, shedding of NRG2 ectodomain by ADAM10 or BACE2 leads to generation of C-terminal fragment (CTF) that remains anchored to the cell membrane; such a fragment may serve as a substrate to γ-secretase. Due to the rapid turnover of CTF, usually this fragment is detectable only upon γ-secretase inhibition [35, 36]. To test whether NRG2, similarly to NRG1, is cleaved by γ-secretase, we treated B16F10 and MC38CEA cells expressing C-terminally HA-tagged NRG2ΔC or NRG1 with one of two γ-secretase inhibitors DAPT or deshydroxy-LY411575. As in the case of NRG1 (both types I and III), Western blotting analysis of NRG2ΔC cleavage products revealed accumulation of NRG2ΔC CTF in lysates of the cells treated with γ-secretase inhibitors (Fig. 6a, Supplementary Fig. 3a). Interestingly, NRG2ΔC CTF was detected as double bands of apparent molecular masses slightly above and below 40 kDa. Incubation of B16F10 cells with DAPT together with any of the metalloprotease inhibitors reduced the amount of NRG2ΔC CTF, but did not significantly change the proportion between the two band densities (Fig. 6b). MC38CEA cells do not express BACE2 and NRG2ΔC CTF in this cell line was also detected as two bands. Treatment of MC38CEA cells with any of the metalloprotease inhibitors including ADAM10-specific inhibitor almost completely abrogated formation of NRG2ΔC CTF (Supplementary Fig. 3b). Therefore, we concluded that the presence of two bands was not a result of cleavage of NRG2ΔC by two different proteases. There was a possibility that a single protease was able to cleave NRG2ΔC at different positions. On the other hand, NRG2ΔC CTF might also undergo post-translational modification. Because most part of NRG2ΔC CTF is located in the cytoplasm and the differences in molecular masses of its two forms are small, we inferred that the most likely modification of CTF is phosphorylation. Indeed, in vitro protein dephosphorylation with alkaline phosphatase led to CTF migrating as a single band with apparent molecular mass lower than any form of untreated CTF (Fig. 6c). Therefore, both forms of NRG2ΔC CTF are phosphorylated, albeit to a different degree. NRG2ΔC CTF is most likely phosphorylated on serine or threonine residues, because we do not routinely add phosphatase inhibitors to the lysis buffer but activity of serine/threonine phosphatases is blocked with EDTA, which is typically present in our lysis buffer. Omission of EDTA in the lysis buffer led, similarly to in vitro dephosphorylation, to detection of NRG2ΔC CTF as a single band (data not shown). In MEF cells overexpressing NRG2 with a full C-terminal sequence, NRG2 CTF was also observed as multiple bands (data not shown).

### Analysis of NRG2 Protein Glycosylation

**Fig. 7:**
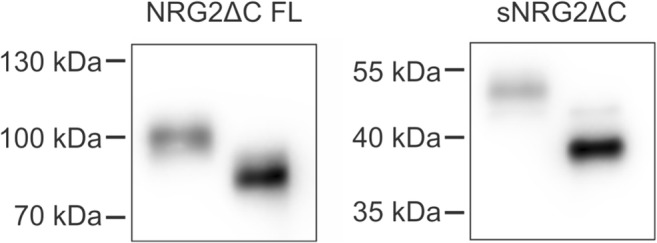
Western blotting analysis of NRG2ΔC glycosylation. Proteins from MEF cell lysates or from concentrated cell culture media were treated with peptide N-glycosidase F (PNGase F), separated by SDS-PAGE and immunoblotted with anti-HA antibody in the case of full-length NRG2ΔC (NRG2ΔC FL) or anti-FLAG antibody in the case of soluble NRG2ΔC (sNRG2ΔC). Representative images from three independent experiments are shown

Predicted molecular mass of NRG2, including both FLAG- and HA-tags, deduced from its translated nucleotide sequence is 79 kDa. However, its apparent molecular mass in SDS-PAGE is about 99 kDa (Fig. 7). We routinely observed NRG2 ectodomain to migrate as several bands, suggesting that it may undergo differential glycosylation. Removal of N-linked glycans with PNGase F from proteins in lysates of MEF cells resulted in 14 kDa downward shift in Western blotting; removal of N-linked glycans from proteins in cell culture medium produced one band corresponding to NRG2 with apparent molecular mass of 38 kDa, 13 kDa less than its glycosylated form. Mouse NRG2 has four potential N-glycosylation sites. Assuming that the molecular mass of a mammalian N-glycan is approximately 2–3.5 kDa depending on a number of antennae, it seems that all of NRG2 N-glycosylation sites may be occupied. Our data show that N-linked protein glycosylation contributes significantly to the discrepancy between the observed molecular mass of NRG2 and the one predicted based on its amino acid sequence.

## Discussion

It is generally accepted that NRGs exert most of their functions by binding to and activating ErbB receptors. Most of NRG isoforms are synthesized as transmembrane proteins and require proteolytic cleavage to liberate biologically active ectodomains. In order to determine which proteases are involved in proteolytic processing of NRG2, we developed cell lines expressing NRG2. Since antibodies specifically recognizing mouse NRG2, suitable for western blotting, are unavailable (we tested several commercially available antibodies and all of them failed in recognizing NRG2), we inserted common tags into coding sequence of NRG2. Introduction of FLAG-tag into several positions of NRG2 ectodomain resulted in protein fragmentation (data not shown). This process was most likely spontaneous owing to the fact that NRG2 fragmentation was not blocked with inhibitors of any class of proteases: AEBSF, PMSF, aprotinin, pepstatin A, E-64, EDTA, or Halt Protease Inhibitor Cocktail. Moreover, the process did not depend on the sequence of an introduced tag, because the insertion of the myc-tag had the same results. The only position that did not interfere with NRG2 biosynthesis was between Ig-like and EGF-like domains.

Vullhorst et al. have shown previously that upon activation of NMDAR subtype of glutamate receptors in primary neurons, rat NRG2 is subject to ectodomain shedding by an enzyme that belongs to metalloproteinases [22]. They also tested the ability of BACE to process NRG2 under these circumstances and concluded that beta-secretase activity is not responsible for rat NRG2 proteolysis. However, the inhibitor used in the study, beta-secretase inhibitor-IV, binds to and blocks activity of BACE1, and not BACE2. Moreover, it was not shown whether rat hippocampal neurons and PC12 cells used in that study expressed BACE1 and/or BACE2; therefore, their data are in line with our results confirming that BACE1 is not a major protease involved in NRG2 processing. In our study, the involvement of BACE1 in NRG2 proteolysis was observed only for NRG2ΔC form and when BACE1 was present in large quantity due to overexpression. Notwithstanding, its ability to shed NRG2 was substantially weaker than that observed for ADAM10 or BACE2.

In their recent work, published during the revision of our manuscript, Vullhorst and Buonanno pointed to ADAM10 as a major sheddase involved in NMDAR-mediated NRG2 processing [37]. NMDAR are nonselective cations channels, highly permeable to calcium ions. Given the fact that ADAM10 is ubiquitously expressed in the central nervous system of adult mice [38, 39] and calcium influx is known activator of ADAM10, NMDAR agonist-stimulated increase in calcium concentration may result in enhanced ADAM10 activity, leading to rapid NRG2 shedding. However, the results presented by Vullhorst and Buonanno [37] may suggest that ADAM10 is not the only protease responsible for NRG2 shedding, because inhibition of ADAM10 (and metalloproteases in general) had less profound effect on NRG2 shedding than NMDAR antagonist. Could BACE2 be this unidentified protease? Unfortunately, little is known about mechanisms underlying regulation of BACE2 activity and it is not recognized whether calcium influx triggered by NMDAR activation may have any impact on it. Although analysis of BACE2 expression in human and rat tissues revealed that most of the brain regions expressed low level of *Bace2* mRNA [40], a recent study indicated that BACE2 is present in a specific subsets of the neurons and glial cells of the mouse brain [41]. Furthermore, BACE2 expression was reported to increase in the brains of aged vs young mice [42]. Hence, BACE2 may be physiologically relevant protease responsible for liberation of NRG2 ectodomain in the adult brain, where NRG2 is primarily expressed. Here, we also confirmed that BACE2 is expressed in the mouse CNS and in some areas, especially in the cerebellar deep nuclei and medulla oblongata, BACE2-expressing cells are also immunoreactive for NRG2. Additionally, hippocampal neurons, isolated from adult mice, showed inhibition of NRG2 shedding after treatment with BACE inhibitor. However, due to generally low expression of endogenous NRG2, we transduced neurons with lentiviral vectors coding for NRG2 under neuron-specific promoter. Therefore, these results may not fully reflect the physiological situation and thus the in vivo significance of BACE2-mediated NRG2 shedding require further studies.

Here we show that NRG2 is efficiently cleaved by either ADAM10 (alpha-secretase) or BACE2 and the effect of inhibition of their activities on NRG2 processing is additive. It supports the hypothesis of competitive ectodomain shedding by alpha- and beta-secretases that was observed for NRG1 and APP processing [18, 43]. It is currently not known, whether NRG2 ectodomains liberated by ADAM10 and BACE2 serve the same function. For example, it has been shown that inhibition of BACE1-mediated NRG1 shedding impaired myelination in an in vitro myelination model, but reduction of NRG1 processing by ADAM10 did not produce the same effect [18]. Moreover, another group have shown that ADAM17, which is also able to cleave NRG1, had an inhibitory effect on myelination in peripheral nervous system [44], possibly via inactivation of NRG1 due to cleavage within its EGF-like domain.

Although NRG2 shedding was severely impaired in ADAM10-deficient MEF cells, we were still able to detect small amounts of soluble NRG2 in the conditioned medium. ADAM10^−/−^ fibroblasts express minute amount of *Bace2* mRNA that is barely detectable using RT-qPCR. Addition of general BACE inhibitor did not eliminate residual NRG2 shedding (Supplementary Fig. 4, 5) therefore endogenous BACE1 or BACE2 is not solely responsible for the liberation of soluble NRG2 from ADAM10^−/−^ MEF cells. Additionally, NRG2 proteolysis was diminished by metalloprotease inhibitors (Supplementary Fig. 4, 5). Hence, additional metalloproteases involved in NRG2 cleavage in ADAM10-deficient cells are yet to be determined.

The NRG2 CTF, which is generated by ectodomain shedding mediated by ADAM10 or BACEs, may serve as a substrate for γ-secretase. It is in agreement with a general opinion that type I membrane protein-derived CTFs are further cleaved by γ-secretase, leading to the release of their intracellular domains (ICDs). These ICDs may serve as transcription regulators, as it was demonstrated for Notch proteins [45] and NRG1 [46, 47]; it is also postulated that regulated intramembrane proteolysis serve as a pathway to facilitate the degradation of membrane proteins whose activity are exerted through their soluble ectodomains [48]. Recent study suggests that NRG2 may have a dual role: in the formation of GABAergic synapses, mediated by its ectodomain, and in the maturation of glutamatergic synapses as a result of NRG2-ICD reverse signaling [49]. It raises an intriguing question whether the effects of NRG2 depletion in mice are a result of lack of its ectodomain or rather signaling-competent ICD.

In summary, we have shown here that murine NRG2 is cleaved near its transmembrane domain by ADAM10 and BACE2. The NRG2 ectodomain shedding leads to the formation of NRG2 CTF, which is then processed by γ-secretase. Another, yet uncharacterized metalloproteases may partially compensate for loss of ADAM10 and/or BACE2, as in ADAM10-deficient cells that also produce negligible amounts of BACEs, NRG2 ectodomain is, although inefficiently, released into extracellular space.

## Electronic Supplementary Material

ESM 1 (PDF 1631 kb)

## References

[1] Carraway KL, Weber JL, Unger MJ (1997). Neuregulin-2, a new ligand of ErbB3/ErbB4-receptor tyrosine kinases. Nature.

[2] Higashiyama S, Horikawa M, Yamada K, Ichino N, Nakano N, Nakagawa T, Miyagawa J, Matsushita N, Nagatsu T, Taniguchi N, Ishiguro H (1997). A novel brain-derived member of the epidermal growth factor family that interacts with ErbB3 and ErbB4. J Biochem (Tokyo).

[3] Hobbs SS, Coffing SL, Le AT (2002). Neuregulin isoforms exhibit distinct patterns of ErbB family receptor activation. Oncogene.

[4] Busfield SJ, Michnick DA, Chickering TW (1997). Characterization of a neuregulin-related gene, Don-1, that is highly expressed in restricted regions of the cerebellum and hippocampus. Mol Cell Biol.

[5] Yan L, Shamir A, Skirzewski M, Leiva-Salcedo E, Kwon OB, Karavanova I, Paredes D, Malkesman O, Bailey KR, Vullhorst D, Crawley JN, Buonanno A (2018). Neuregulin-2 ablation results in dopamine dysregulation and severe behavioral phenotypes relevant to psychiatric disorders. Mol Psychiatry.

[6] Meyer D, Birchmeier C (1995). Multiple essential functions of neuregulin in development. Nature.

[7] Gassmann M, Casagranda F, Orioli D, Simon H, Lai C, Klein R, Lemke G (1995). Aberrant neural and cardiac development in mice lacking the ErbB4 neuregulin receptor. Nature.

[8] Lee K-F, Simon H, Chen H, Bates B, Hung MC, Hauser C (1995). Requirement for neuregulin receptor erbB2 in neural and cardiac development. Nature.

[9] Erickson SL, O’Shea KS, Ghaboosi N (1997). ErbB3 is required for normal cerebellar and cardiac development: a comparison with ErbB2-and heregulin-deficient mice. Development.

[10] Britto JM, Lukehurst S, Weller R, Fraser C, Qiu Y, Hertzog P, Busfield SJ (2004). Generation and characterization of neuregulin-2-deficient mice. Mol Cell Biol.

[11] Mostaid MS, Mancuso SG, Liu C, Sundram S, Pantelis C, Everall IP, Bousman CA (2017). Meta-analysis reveals associations between genetic variation in the 5′ and 3′ regions of neuregulin-1 and schizophrenia. Transl Psychiatry.

[12] Mostaid MS, Lloyd D, Liberg B, Sundram S, Pereira A, Pantelis C, Karl T, Weickert CS, Everall IP, Bousman CA (2016). Neuregulin-1 and schizophrenia in the genome-wide association study era. Neurosci Biobehav Rev.

[13] Benzel I, Bansal A, Browning BL, Galwey NW, Maycox PR, McGinnis R, Smart D, St Clair D, Yates P, Purvis I (2007). Interactions among genes in the ErbB-Neuregulin signalling network are associated with increased susceptibility to schizophrenia. Behav Brain Funct.

[14] Schwab SG, Eckstein GN, Hallmayer J, Lerer B, Albus M, Borrmann M, Lichtermann D, Ertl MA, Maier W, Wildenauer DB (1997). Evidence suggestive of a locus on chromosome 5q31 contributing to susceptibility for schizophrenia in German and Israeli families by multipoint affected sib-pair linkage analysis. Mol Psychiatry.

[15] Sklar P, Pato MT, Kirby A, Petryshen TL, Medeiros H, Carvalho C, Macedo A, Dourado A, Coelho I, Valente J, Soares MJ, Ferreira CP, Lei M, Verner A, Hudson TJ, Morley CP, Kennedy JL, Azevedo MH, Lander E, Daly MJ, Pato CN (2004). Genome-wide scan in Portuguese Island families identifies 5q31–5q35 as a susceptibility locus for schizophrenia and psychosis. Mol Psychiatry.

[16] Straub RE, MacLean CJ, O’Neill FA (1997). Support for a possible schizophrenia vulnerability locus in region 5q22–31 in Irish families. Mol Psychiatry.

[17] Arcos-Burgos M, Castellanos FX, Pineda D, Lopera F, Palacio JD, Palacio LG, Rapoport JL, Berg K, Bailey-Wilson JE, Muenke M (2004). Attention-deficit/hyperactivity disorder in a population isolate: linkage to loci at 4q13.2, 5q33.3, 11q22, and 17p11. Am J Hum Genet.

[18] Luo X, Prior M, He W, Hu X, Tang X, Shen W, Yadav S, Kiryu-Seo S, Miller R, Trapp BD, Yan R (2011). Cleavage of neuregulin-1 by BACE1 or ADAM10 protein produces differential effects on myelination. J Biol Chem.

[19] Montero JC, Yuste L, Díaz-Rodríguez E (2000). Differential shedding of transmembrane neuregulin isoforms by the tumor necrosis factor-α-converting enzyme. Mol Cell Neurosci.

[20] Fleck D, van Bebber F, Colombo A, Galante C, Schwenk BM, Rabe L, Hampel H, Novak B, Kremmer E, Tahirovic S, Edbauer D, Lichtenthaler SF, Schmid B, Willem M, Haass C (2013). Dual cleavage of neuregulin 1 type III by BACE1 and ADAM17 liberates its EGF-like domain and allows paracrine signaling. J Neurosci.

[21] Shirakabe K, Wakatsuki S, Kurisaki T, Fujisawa-Sehara A (2001). Roles of Meltrin β/ADAM19 in the processing of neuregulin. J Biol Chem.

[22] Vullhorst D, Mitchell RM, Keating C, Roychowdhury S, Karavanova I, Tao-Cheng JH, Buonanno A (2015). A negative feedback loop controls NMDA receptor function in cortical interneurons via neuregulin 2/ErbB4 signalling. Nat Commun.

[23] Bereta M, Hayhurst A, Gajda M, Chorobik P, Targosz M, Marcinkiewicz J, Kaufman HL (2007). Improving tumor targeting and therapeutic potential of Salmonella VNP20009 by displaying cell surface CEA-specific antibodies. Vaccine.

[24] Chomczynski P (1987). Single-step method of RNA isolation by acid guanidinium thiocyanate–phenol–chloroform extraction. Anal Biochem.

[25] Pritlove DC, Poon LL, Fodor E, Sharps J, Brownlee GG (1998). Polyadenylation of influenza virus mRNA transcribed in vitro from model virion RNA templates: requirement for 5′ conserved sequences. J Virol.

[26] Laible M, Boonrod K (2009) Homemade site directed mutagenesis of whole plasmids. J Vis Exp. 10.3791/1135 10.3791/1135 PMC2762917 19488024

[27] Weber K, Bartsch U, Stocking C, Fehse B (2008). A multicolor panel of novel lentiviral “gene ontology” (LeGO) vectors for functional gene analysis. Mol Ther.

[28] Kowarz E, Löscher D, Marschalek R (2015). Optimized sleeping beauty transposons rapidly generate stable transgenic cell lines. Biotechnol J.

[29] Czarnek M, Bereta J (2017). SmartFlares fail to reflect their target transcripts levels. Sci Rep.

[30] Mátés L, Chuah MKL, Belay E, Jerchow B, Manoj N, Acosta-Sanchez A, Grzela DP, Schmitt A, Becker K, Matrai J, Ma L, Samara-Kuko E, Gysemans C, Pryputniewicz D, Miskey C, Fletcher B, VandenDriessche T, Ivics Z, Izsvák Z (2009). Molecular evolution of a novel hyperactive Sleeping Beauty transposase enables robust stable gene transfer in vertebrates. Nat Genet.

[31] Schindelin J, Arganda-Carreras I, Frise E, Kaynig V, Longair M, Pietzsch T, Preibisch S, Rueden C, Saalfeld S, Schmid B, Tinevez JY, White DJ, Hartenstein V, Eliceiri K, Tomancak P, Cardona A (2012). Fiji: an open-source platform for biological-image analysis. Nat Methods.

[32] Brewer GJ, Torricelli JR (2007). Isolation and culture of adult neurons and neurospheres. Nat Protoc.

[33] Ludwig A, Hundhausen C, Lambert M, Broadway N, Andrews RC, Bickett DM, Leesnitzer MA, Becherer JD (2005). Metalloproteinase inhibitors for the disintegrin-like metalloproteinases ADAM10 and ADAM17 that differentially block constitutive and phorbol ester-inducible shedding of cell surface molecules. Comb Chem High Throughput Screen.

[34] Longart M, Liu Y, Karavanova I, Buonanno A (2004). Neuregulin-2 is developmentally regulated and targeted to dendrites of central neurons. J Comp Neurol.

[35] Carey BW, Kim DY, Kovacs DM (2007). Presenilin/gamma-secretase and alpha-secretase-like peptidases cleave human MHC class I proteins. Biochem J.

[36] Haas IG, Frank M, Veron N, Kemler R (2005). Presenilin-dependent processing and nuclear function of -protocadherins. J Biol Chem.

[37] Vullhorst D, Buonanno A (2019). NMDA receptors regulate neuregulin 2 binding to ER-PM junctions and ectodomain release. Mol Neurobiol.

[38] Guo Z-B, Su Y-Y, Wang Y-H, Wang W, Guo DZ (2016). The expression pattern of Adam10 in the central nervous system of adult mice: detection by in situ hybridization combined with immunohistochemistry staining. Mol Med Rep.

[39] Endres K, Deller T (2017) Regulation of alpha-secretase ADAM10 in vitro and in vivo: genetic, epigenetic, and protein-based mechanisms. Front Mol Neurosci 10. 10.3389/fnmol.2017.00056 10.3389/fnmol.2017.00056 PMC5355436 28367112

[40] Bennett BD, Babu-Khan S, Loeloff R, Louis JC, Curran E, Citron M, Vassar R (2000). Expression analysis of BACE2 in brain and peripheral tissues. J Biol Chem.

[41] Voytyuk I, Mueller SA, Herber J, Snellinx A, Moechars D, van Loo G, Lichtenthaler SF, de Strooper B (2018). BACE2 distribution in major brain cell types and identification of novel substrates. Life Sci Alliance.

[42] Wang Z, Xu Q, Cai F (2019). BACE2, a conditional β-secretase, contributes to Alzheimer’s disease pathogenesis. JCI Insight.

[43] Skovronsky DM, Moore DB, Milla ME, Doms RW, Lee VM (2000). Protein kinase C-dependent α-secretase competes with β-secretase for cleavage of amyloid-β precursor protein in the trans-Golgi network. J Biol Chem.

[44] La Marca R, Cerri F, Horiuchi K (2011). TACE (ADAM17) inhibits Schwann cell myelination. Nat Neurosci.

[45] Andersson ER, Sandberg R, Lendahl U (2011). Notch signaling: simplicity in design, versatility in function. Development.

[46] Bao J, Wolpowitz D, Role LW, Talmage DA (2003). Back signaling by the Nrg-1 intracellular domain. J Cell Biol.

[47] Chen Y, Hancock ML, Role LW, Talmage DA (2010). Intramembranous valine linked to schizophrenia is required for neuregulin 1 regulation of the morphological development of cortical neurons. J Neurosci.

[48] Kopan R, Ilagan MXG (2004). γ-Secretase: proteasome of the membrane?. Nat Rev Mol Cell Biol.

[49] Lee K-H, Lee H, Yang CH, Ko JS, Park CH, Woo RS, Kim JY, Sun W, Kim JH, Ho WK, Lee SH (2015). Bidirectional signaling of neuregulin-2 mediates formation of GABAergic synapses and maturation of glutamatergic synapses in newborn granule cells of postnatal hippocampus. J Neurosci.

