# Supplementary material for: Proteolytic Processing of Neuregulin 2
Source: Mol Neurobiol. 2019 Dec 14;57(4):1799–813. doi: 10.1007/s12035-019-01846-9 (PMC7118043; doi:10.1007/s12035-019-01846-9)

Proteolytic processing of neuregulin 2

Maria Czarnek[1], Joanna Bereta[1]

[1]Department of Cell Biochemistry, Faculty of Biochemistry, Biophysics and Biotechnology, Jagiellonian University in Kraków, Gronostajowa 7, 30-387 Kraków, Poland

Corresponding authors: Maria Czarnek, e-mail: maria.czarnek@doctoral.uj.edu.pl; Joanna Bereta, e-mail: joanna.bereta@uj.edu.pl

Supplementary materials and methods

Immunofluorescence Cells were transfected with plasmids coding for NRG2 or one of BACEs or corresponding empty vector. One day after transfection, the cells were seeded on poly-L-lysine-coated coverslips. On the following day, the cells were washed with PBS and fixed in solution containing 4% formaldehyde and 4% sucrose in PBS for 15 min at room temperature. After TBS washes, the cells were permeabilized with 0.1% Triton X-100 in TBS for 10 min at room temperature, washed in TBS and incubated with blocking solution (10% normal goat serum, 1% BSA in TBST) for 1 h at room temperature, then incubated with primary antibody: mouse anti-NRG2 or rabbit anti-BACE2 overnight at 4°C. After 3 washes with TBST, the cells were incubated with secondary antibody: Alexa Fluor 488 anti-mouse IgG or anti-rabbit IgG (both from Thermo Scientific, 1:500) for 1 h at room temperature, followed by 3 washes in TBST, brief wash in distilled water and mounting with Prolong Glass with NucBlue (Thermo Scientific). After overnigth curing, slides were imaged on Leica DMIL LED microscope.

Supplementary Table 1 List of primers used for cloning

	Forward primer	Reverse primer
NRG1 type I	ATGTCTGAGCGCAAAGAAGG	TTATACAGCAATAGGGTCTTGGT
NRG1 type III	ATGGAG ATTTAT CCCCCA GAC	TTATACAGCAATAGGGTCTTGGT
NRG2	ATGCGGCAGGTTTGCTGCTC	TTAGAGGGGCCCCGAGTCCT
NRG1 HA tag (inverse PCR)	CATCATATGGATATACAGCAATAG GGT	TTCCAGATTATGCTTAAATCTTCT AGAAG
NRG2 HA tag (Quikchange)	TCGCTGTCATTACCCATACGATGT TCCAGATTACGCTTGAGCAGCGG	CCGCTGCTCAAGCGTAATCTGGAA CATCGTATGGGTAATGACAGCGA
NRG2 FLAG tag (inverse PCR)	GATGACGACAAGGGACATGCCCG GAAGTG	GTCTTTGTAGTCCGACCAGGATGA CAGAGTG
*Bam*HI/*Not*I ADAM10	GGATCCATGGTGTTGCCCACAGTG	GCGGCCGCTTAGCGTCGCATG
*Eco*RI/*Not*I BACE1	GAATTCATGGCCCCAGCGCTGCAC TG	GCGGCCGCTTACTTGAGCAGGGA GATGT
*Bam*HI/*Eco*RI BACE2	ATAGGATCCATGGGCGCGCTGCTT CGA	GCGCGAATTCTCATTTCCAGCGAT GTCTGACTA
*Sfi*I NRG2/HA tag	AGGCCTCTGAGGCCACCATGCGG CAGGTTTGCTGCTC	GCGGCCTGACAGGCCTTAAGCGT AATCTGGAACATCG

Supplementary Table 2 List of primers used for RT-qPCR

Gene	Forward primer	Reverse primer
Ef2	CCACGGCAAGTCCACGCTGAC	AGAAGAGGGAGATGGCGGTGGATT
Tbp	CAGTGCCCAGCATCACTATTTC	AAGCCCTGAGCATAAGGTGG
PolR2b	GGATTCTGGGAACGTCGGAG	CCGGAGTGATCTCATCGTCG
ActB	ATTACTGCTCTGGCTCCTAGC	CAGCTCAGTAACAGTCCGCCTA
Adam10	CCGGGCTCTCCATGTAATGA	CCAGTGAGCCACAATCCAC
Bace1	GAGAGGCAGCTTTGTGGAGA	CGTGTCCACCAGGATGTTGA
Bace2	CTTTGCAGTGAGTCCCTGTGC	GCAGCAGGAGGAGGATCAGAAC
Nrg2	GTCTGTGAGGCCGAGAACAT	TCGATGTAGTAGCACACGCC

Coding sequence of NRG2ΔC. FLAG- and HA-tags are in bold.

ATGCGGCAGGTTTGCTGCTCAGCGCTGCCGCCGCCACTGGAGAAGGCTCGGTGCAGCAGCTACAGCTACAGTT
ACAGCGACAGCAGCAGCACCACCAGCAGCAGCAGAAGCAGCAGCAGCAGCAGCAGAAACAGCAGCAGCAG
CAACAGCAGCAGCAGCGAGAGCAGCGGCAGCAACAGCGGCAGCAGCAGCATCTTCCGTCCCGCTGCGCCCCCA
GAGCCGCGGCCGCAGCCACAGCCGCAGCCCCGCAGCCCCGCAGCCCGGAGAGCCGCCGCCCGCTCGCGAGCCG
CAGCCGCCGGCGGCATGAGGCGCGACCCGGCCCCCGGCTTCTCGATGCTGCTCTTCGGTGTGTCACTCGCCTG
CTACTCGCCCAGCCTCAAGTCGGTGCAGGACCAGGCGTACAAGGCACCCGTGGTGGTGGAGGGCAAGGTACAG
GGACTGGCCCCGGCAGGCGGTTCCAGCTCTAACAGCACCCGAGAGCCGCCCGCCTCGGGTCGGGTGGCGCTGG
TGAAGGTGCTGGACAAGTGGCCGCTCCGGAGCGGGGGGCTGCAGCGCGAGCAGGTGATCAGCGTGGGCTCCTG
TGCGCCGCTCGAAAGGAACCAGCGCTACATCTTTTTCCTGGAGCCCACCGAGCAGCCCTTAGTTTTTAAGACA
GCCTTTGCCCCGGTCGACCCTAACGGCAAAAACATCAAGAAAGAGGTGGGCAAGATCCTGTGCACTGACTGCG
CCACCCGGCCCAAGCTGAAGAAGATGAAGAGCCAGACAGGAGAGGTGGGTGAGAAGCAGTCGCTCAAGTGTGA
GGCAGCGGCGGGAAACCCCCAGCCCTCCTATCGCTGGTTCAAGGATGGCAAGGAACTCAACCGGAGTCGTGAT
ATTCGCATCAAGTATGGCAATGGCAGAAAGAACTCACGGCTACAGTTCAACAAAGTGAGGGTGGAGGATGCCG
GGGAGTACGTCTGTGAGGCCGAGAACATCCTTGGGAAGGACACCGTGAGGGGCCGACTCCATGTCAACAGCGT
GAGCACCACTCTGTCATCCTGGTCG**GACTACAAAGACGATGACGACAAG**GGACATGCCCGGAAGTGCAATGAG
ACCGCCAAGTCCTACTGTGTGAATGGAGGCGTGTGCTACTACATCGAGGGCATCAACCAGCTCTCCTGCAAGT
GTCCTGTGGGATACACCGGGGACAGGTGTCAGCAGTTCGCAATGGTCAACTTCTCCAAGCACCTTGGATTTGA
ATTGAAGGAGGCTGAGGAGCTGTACCAGAAGAGAGTGCTGACAATTACTGGTATCTGTGTGGCCCTGCTGGTC
GTGGGCATCGTCTGTGTGGTCGCCTACTGCAAGACCAAAAAACAGAGGAGGCAGATGCATCATCATCTCCGGC
AGAACATGTGCCCAGCCCACCAGAACCGAAGCCTGGCCAACGGGCCCAGCCACCCTCGGCTGGACCCTGAGGA
GATCCAGATGGCAGATTACATCTCCAAAAATGTGCCAGCTACAGACCACGTGATCCGGAGGGAAGCTGAGACC
ACGTTCTCTGGGAGCCACTCCTGTTCACCTTCTCACCACTGCTCCACAGCCACGCCCACCTCCAGCCACAGAC
ATGAGAGCCACACGTGGAGCCTGGAACGTTCAGAGAGCCTGACCTCGGATTCCCAGTCAGGCATCATGCTATC
ATCAGTAGGCACCAGCAAGTGCAACAGCCCAGCATGTGTGGAGGCACGGGCGCGGAGGGCAGCAGCCTACAGC
CAGGAGGAGCGGCGCAGGGCTGCCATGCCACCCTACCATGACTCCATAGACTCGCTGCGTGACTCTCCACACA
GTGAAAGGTACGTGTCAGCCTTGACCACGCCCGCTCGCCTCTCGCCCGTGGACTTCCACTACTCGCTGGCCAC
GCAGGTGCCGACTTTCGAGATCACGTCGCCCAACTCTGCGCATGCCGTGTCGCTGCCGCCCGCCGCGCCCATC
AGCTACCGCCTGGCGGAGCAGCAGCCGCTCCTGCGGCATCCAGCGCCGCCCGGCCCGGGGCCGGGGTCGGGGC
CCGGAGCGGACATGCAGCGCAGCTACGACAGCTACTACTACCCTGCGGCGGGGCCCGGGCCGCGGCGCAGCGC
GCACGCGCGGCGCGGGACTCGCTGTCAT**TACCCATACGATGTTCCAGATTACGCT**TGA

Coding sequence of NRG2. FLAG- and HA-tags are in bold, Ser-Gly linker is italicized.

ATGCGGCAGGTTTGCTGCTCAGCGCTGCCGCCGCCACTGGAGAAGGCTCGGTGCAGCAGCTACAGCTACAGTT
ACAGCGACAGCAGCAGCACCACCAGCAGCAGCAGAAGCAGCAGCAGCAGCAGCAGCAGAAACAGCAGCAGCAG
CAACAGCAGCAGCAGCGAGAGCAGCGGCAGCAACAGCGGCAGCAGCAGCATCTTCCGTCCCGCTGCGCCCCCA
GAGCCGCGGCCGCAGCCACAGCCGCAGCCCCGCAGCCCCGCAGCCCGGAGAGCCGCCGCCCGCTCGCGAGCCG
CAGCCGCCGGCGGCATGAGGCGCGACCCGGCCCCCGGCTTCTCGATGCTGCTCTTCGGTGTGTCACTCGCCTG
CTACTCGCCCAGCCTCAAGTCGGTGCAGGACCAGGCGTACAAGGCACCCGTGGTGGTGGAGGGCAAGGTACAG
GGACTGGCCCCGGCAGGCGGTTCCAGCTCTAACAGCACCCGAGAGCCGCCCGCCTCGGGTCGGGTGGCGCTGG
TGAAGGTGCTGGACAAGTGGCCGCTCCGGAGCGGGGGGCTGCAGCGCGAGCAGGTGATCAGCGTGGGCTCCTG
TGCGCCGCTCGAAAGGAACCAGCGCTACATCTTTTTCCTGGAGCCCACCGAGCAGCCCTTAGTTTTTAAGACA
GCCTTTGCCCCGGTCGACCCTAACGGCAAAAACATCAAGAAAGAGGTGGGCAAGATCCTGTGCACTGACTGCG
CCACCCGGCCCAAGCTGAAGAAGATGAAGAGCCAGACAGGAGAGGTGGGTGAGAAGCAGTCGCTCAAGTGTGA
GGCAGCGGCGGGAAACCCCCAGCCCTCCTATCGCTGGTTCAAGGATGGCAAGGAACTCAACCGGAGTCGTGAT
ATTCGCATCAAGTATGGCAATGGCAGAAAGAACTCACGGCTACAGTTCAACAAAGTGAGGGTGGAGGATGCCG
GGGAGTACGTCTGTGAGGCCGAGAACATCCTTGGGAAGGACACCGTGAGGGGCCGACTCCATGTCAACAGCGT
GAGCACCACTCTGTCATCCTGGTCG**GACTACAAAGACGATGACGACAAG**GGACATGCCCGGAAGTGCAATGAG
ACCGCCAAGTCCTACTGTGTGAATGGAGGCGTGTGCTACTACATCGAGGGCATCAACCAGCTCTCCTGCAAGT
GTCCTGTGGGATACACCGGGGACAGGTGTCAGCAGTTCGCAATGGTCAACTTCTCCAAGCACCTTGGATTTGA
ATTGAAGGAGGCTGAGGAGCTGTACCAGAAGAGAGTGCTGACAATTACTGGTATCTGTGTGGCCCTGCTGGTC
GTGGGCATCGTCTGTGTGGTCGCCTACTGCAAGACCAAAAAACAGAGGAGGCAGATGCATCATCATCTCCGGC
AGAACATGTGCCCAGCCCACCAGAACCGAAGCCTGGCCAACGGGCCCAGCCACCCTCGGCTGGACCCTGAGGA
GATCCAGATGGCAGATTACATCTCCAAAAATGTGCCAGCTACAGACCACGTGATCCGGAGGGAAGCTGAGACC
ACGTTCTCTGGGAGCCACTCCTGTTCACCTTCTCACCACTGCTCCACAGCCACGCCCACCTCCAGCCACAGAC
ATGAGAGCCACACGTGGAGCCTGGAACGTTCAGAGAGCCTGACCTCGGATTCCCAGTCAGGCATCATGCTATC
ATCAGTAGGCACCAGCAAGTGCAACAGCCCAGCATGTGTGGAGGCACGGGCGCGGAGGGCAGCAGCCTACAGC
CAGGAGGAGCGGCGCAGGGCTGCCATGCCACCCTACCATGACTCCATAGACTCGCTGCGTGACTCTCCACACA
GTGAAAGGTACGTGTCAGCCTTGACCACGCCCGCTCGCCTCTCGCCCGTGGACTTCCACTACTCGCTGGCCAC
GCAGGTGCCGACTTTCGAGATCACGTCGCCCAACTCTGCGCATGCCGTGTCGCTGCCGCCCGCCGCGCCCATC
AGCTACCGCCTGGCGGAGCAGCAGCCGCTCCTGCGGCATCCAGCGCCGCCCGGCCCGGGGCCGGGGTCGGGGC
CCGGAGCGGACATGCAGCGCAGCTACGACAGCTACTACTACCCTGCGGCTGGCCCCGGACCTAGAAGATCTGC
TTGTGCTCTCGGCGGCAGCCTGGGATCTCTTCCAGCTAGCCCTTTCAGAATCCCCGAGGACGACGAGTACGAG
ACAACCCAAGAGTGTGCCCCTCCACCTCCTCCAAGACCTAGAACAAGAGGCGCCAGCAGAAGAACCAGCGCCG
GACCAAGACGTTGGAGAAGAAGCAGACTGAACGGCCTGGCCGCTCAGAGAGCTAGAGCCGCTAGAGACTCTCT
GTCTCTGAGCAGCGGCTCTGGCTGTGGCTCTGCCTCTGCTTCTGACGACGATGCCGATGATGCTGATGGCGCC
CTGGCTGCTGAGTCTACACCTTTTCTGGGCCTGAGAGCCGCTCACGACGCCCTGAGATCTGATAGCCCTCCTC
TGTGTCCTGCCGCTGACAGCAGAACCTACTACAGCCTGGACAGCCACTCCACCAGAGCCAGCAGCAGACACTC
TAGAGGCCCTCCAACAAGAGCCAAGCAGGACTCTGGACCTCTT*GGCGGCGGAGGATCTGGCGGAGGCGGATCT*
TATCCTTACGACGTGCCAGACTACGCCTGA

a

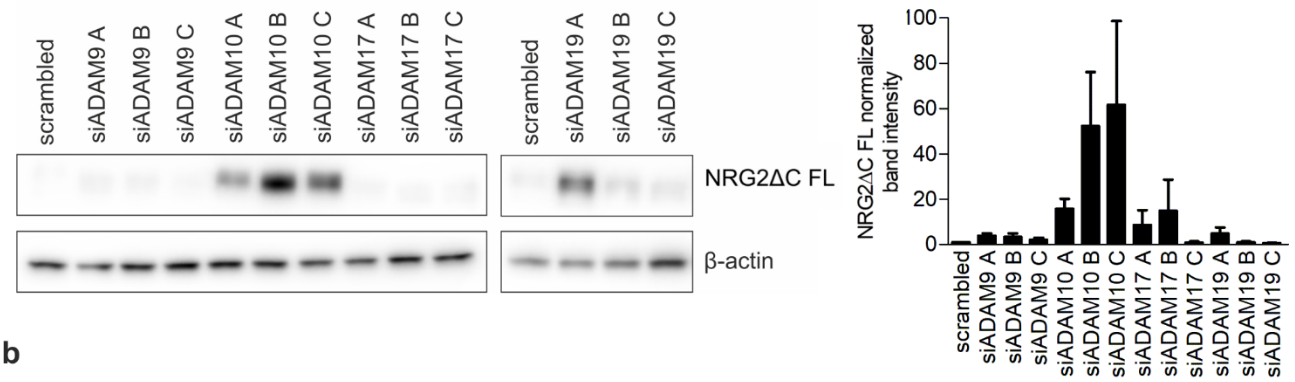

b

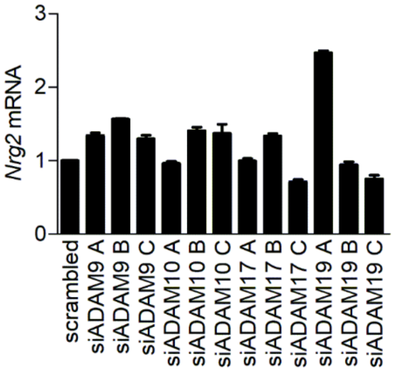

Supplementary Fig. 1 Silencing of ADAM10 expression limits NRG2 shedding. (a) Western blotting analysis of full-length NRG2ΔC in lysates of B16F10 cells transduced with a vector coding for HA-tagged NRG2ΔC upon silencing of ADAM9, ADAM10, ADAM17, or ADAM19 expression using three different siRNA for each transcript. Beta-actin served as a loading control. Right panel: quantification of WB signal. Graph represents the fold change of band intensities normalized to β-actin; band intensity of a sample from the cells transfected with scrambled siRNA is set as 1. Mean values ± SEM from 2 independent experiments are shown. (b) *Nrg2* transcript levels in B16F10 cells transfected with siRNA against ADAM9, ADAM10, ADAM17, or ADAM19. The data shown are mean values ± SD from duplicates from the same transfection as shown in (a).

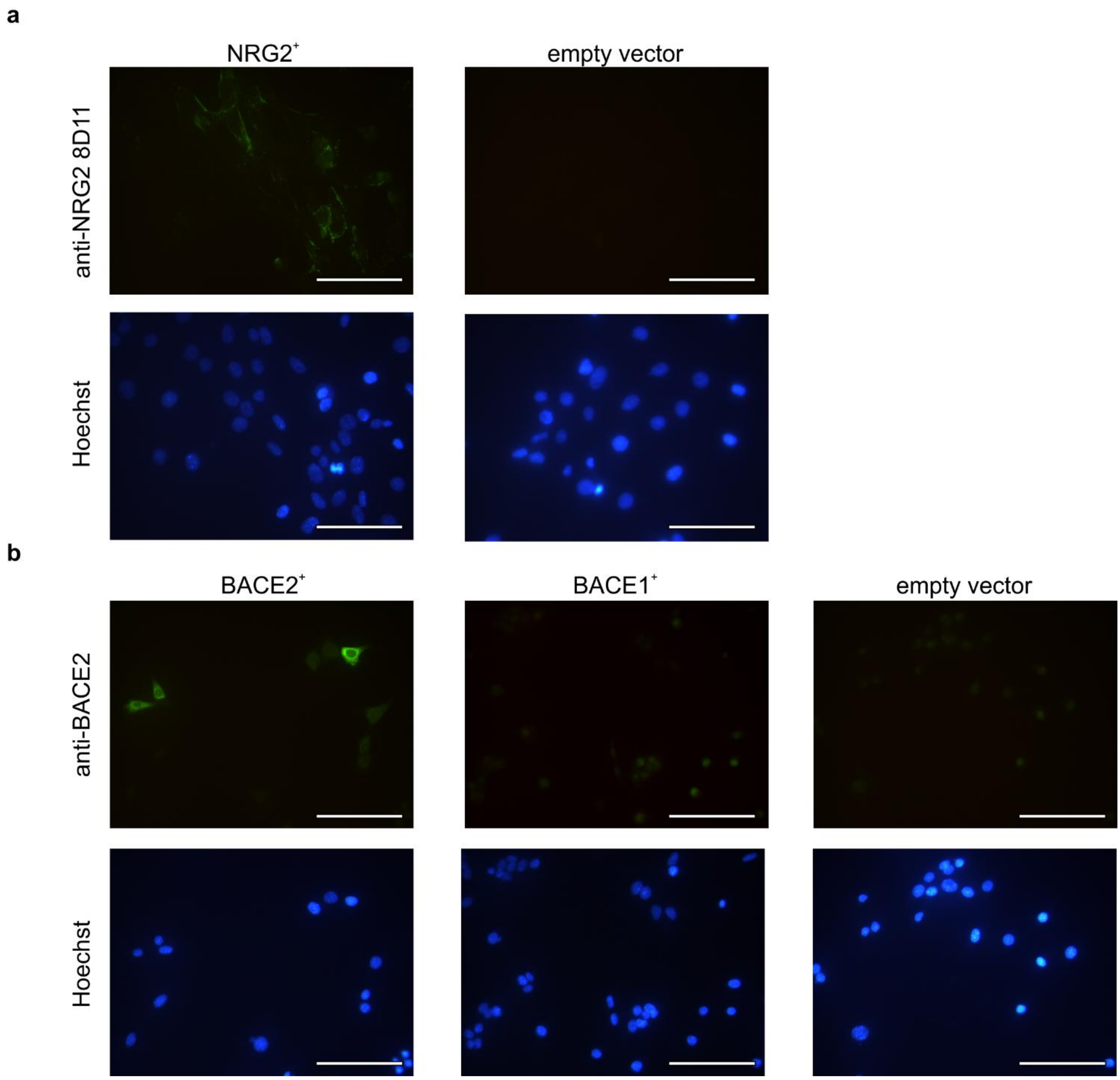

Supplementary Fig. 2 Immunofluorescent staining of (a) NRG2 in MEF ADAM10$^{-/-}$ cells transfected with vector coding for NRG2 or empty vector or (b) BACE2 in MEF cells transfected with vector coding for BACE2 or BACE1, or empty vector. Cell nuclei in the analyzed fields are visualized with Hoechst dye. Scale bar = 100 µm

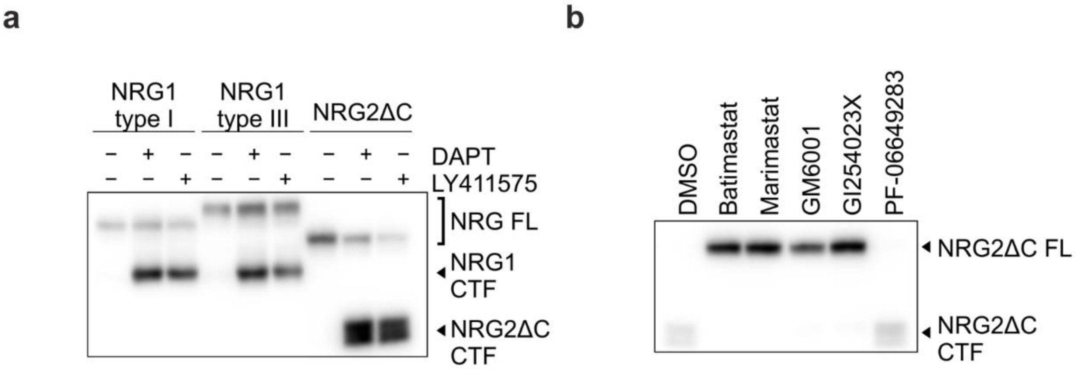

Supplementary Fig. 3 NRG2ΔC is a substrate for γ-secretase. (a) Western blotting analysis of NRG2ΔC C-terminal fragment (CTF) accumulation in MC38CEA cells transduced with C-terminally HA-tagged NRG2ΔC upon **γ**-secretase inhibition with DAPT or deshydroxy-LY411575 (denoted as LY411575). A representative image from 2 experiments is shown. (b) Western blotting analysis of NRG2ΔC C-terminal fragment accumulation in MC38CEA cells transduced with C-terminally HA-tagged NRG2ΔC upon simultaneous inhibition of NRG2ΔC processing with metalloprotease or BACE inhibitor and **γ**-secretase inhibitor (DAPT). Batimastat, Marimastat, and GM6001 are general metalloprotease inhibitors, GI254023X is a specific ADAM10 inhibitor, PF-06649283 – general BACE inhibitor. A representative image from 2 experiments is shown.

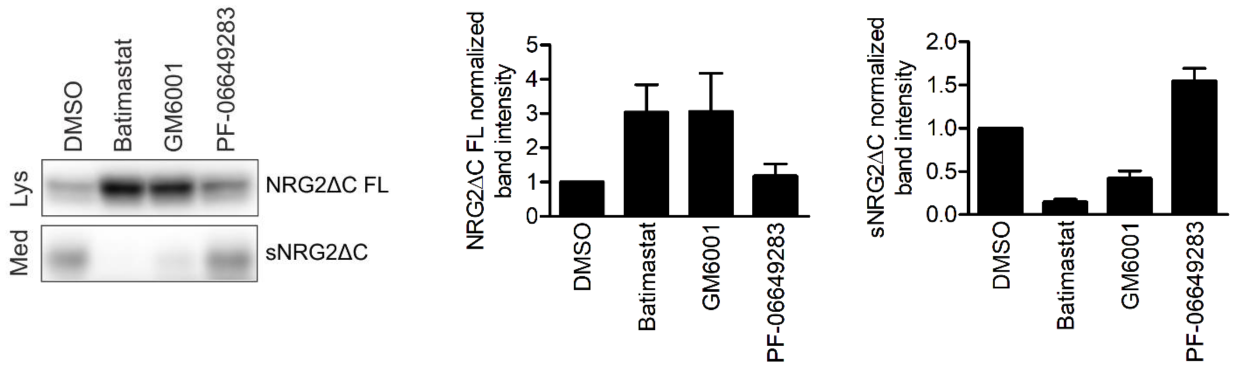

Supplementary Fig. 4 Inhibition of metalloproteases activity, but not BACE activity, limits NRG2ΔC shedding in ADAM10-deficient cells. Western blotting analysis of NRG2ΔC shedding in ADAM10$^{-/-}$ MEF cells transduced with a vector coding for FLAG- and HA-tagged NRG2ΔC upon metalloproteases inhibition with Batimastat or GM6001, or BACE inhibition with general BACE inhibitor PF-06649283. Lys – cell lysates; Med – concentrated cell culture media; NRG2ΔC FL – full length NRG2ΔC, sNRGΔC – soluble NRG2ΔC. Right panel: quantification of WB signal. Graph represents mean values of fold changes of NRG2-linked chemiluminescent signals ± SEM of 3 independent experiments. The chemiluminescent signal of the sample from the cells treated with DMSO (vehicle) was set as 1.

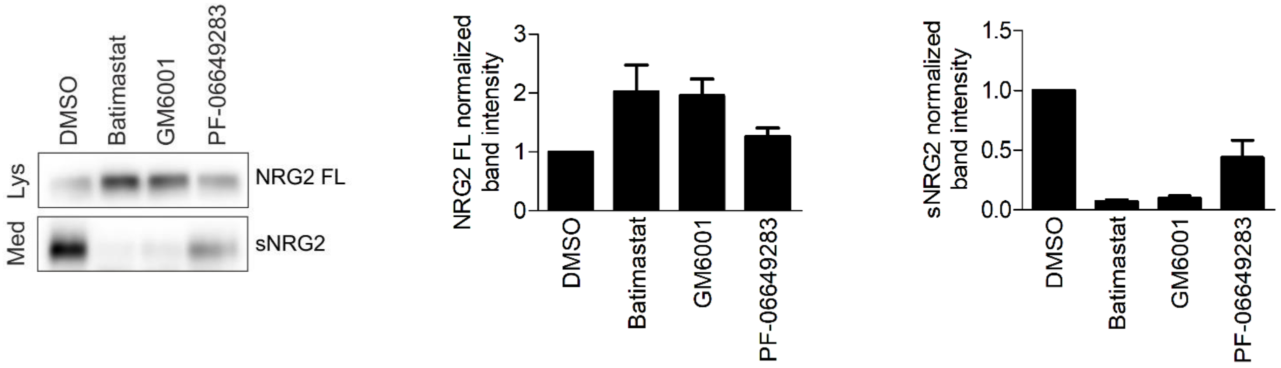

Supplementary Fig. 5 Inhibition of metalloproteases activity or BACE activity, limits shedding of NRG2 with full C-terminal sequence in ADAM10-deficient cells. Western blotting analysis of NRG2 shedding in ADAM10$^{-/-}$ MEF cells transduced with a vector coding for FLAG- and HA-tagged NRG2 full-length C-terminal upon metalloproteases inhibition with Batimastat or GM6001, or BACE inhibition with general BACE inhibitor PF-06649283. Lys – cell lysates; Med – concentrated cell culture media; NRG2 FL – full length NRG2, sNGR2 – soluble NRG2. Right panel: quantification of WB signal. Graph represents mean values of fold changes of NRG2-linked chemiluminescent signals ± SEM of 3 independent experiments. The chemiluminescent signal of the sample from the cells treated with DMSO (vehicle) was set as 1.

Uncropped images and pictures of membranes stained with Ponceau S from Fig. 1-7 and Supplementary Fig. 1-5

Figure 1

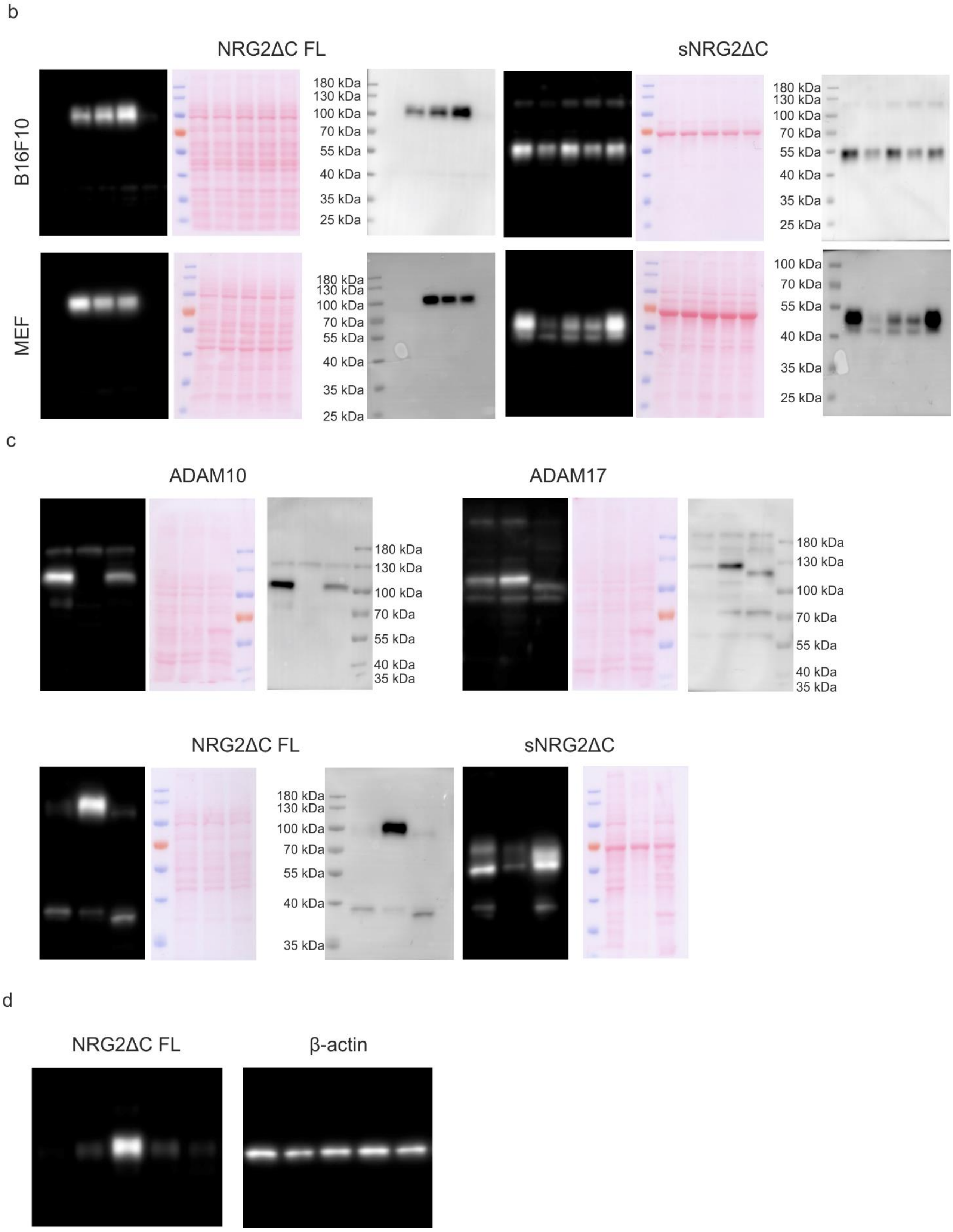

Figure 2

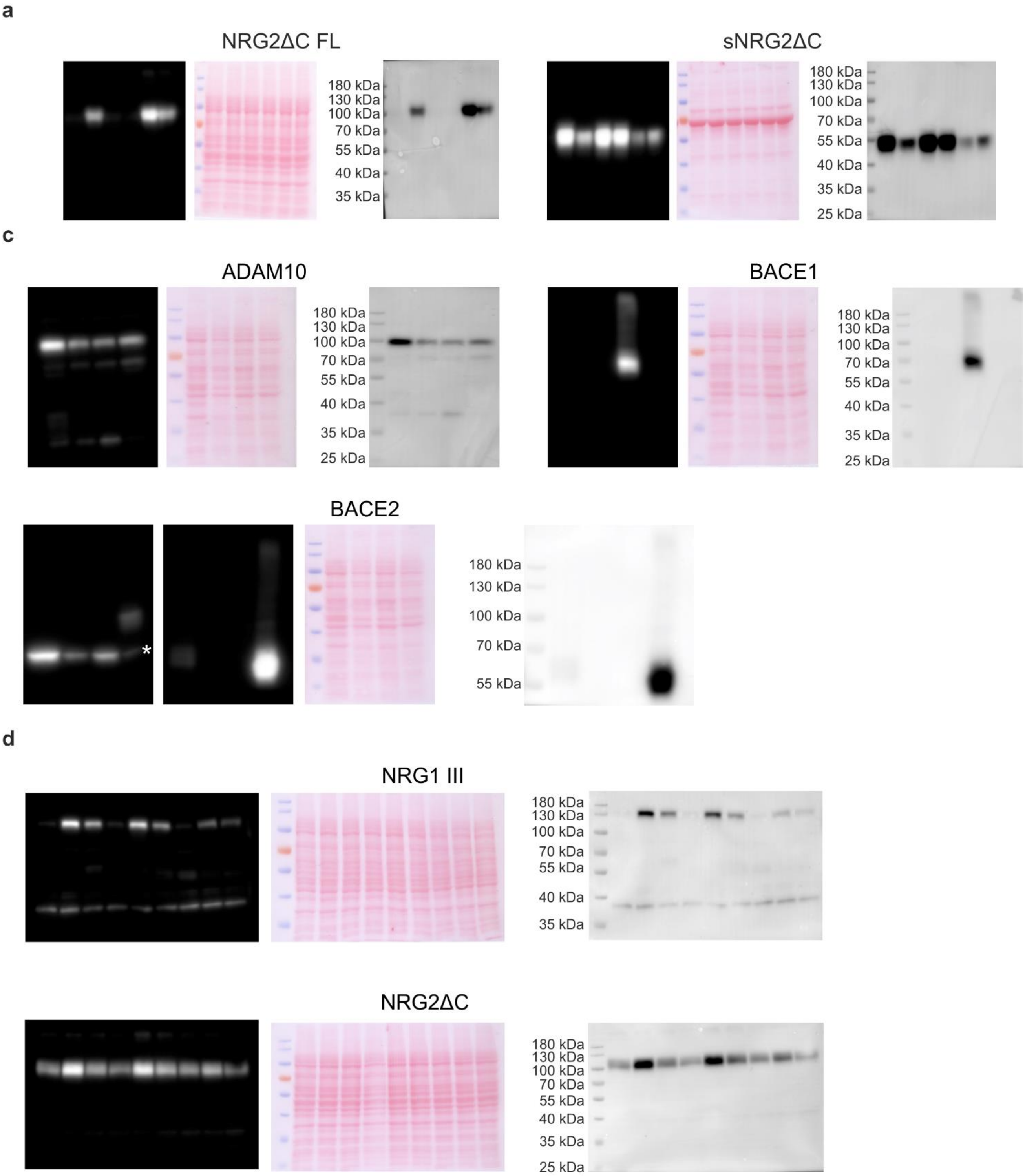
a
NRG2ΔC FL
180 kDa
130 kDa
100 kDa
70 kDa
55 kDa
40 kDa
35 kDa
sNRG2ΔC
180 kDa
130 kDa
100 kDa
70 kDa
55 kDa
40 kDa
35 kDa
25 kDa
c
ADAM10
180 kDa
130 kDa
100 kDa
70 kDa
55 kDa
40 kDa
35 kDa
25 kDa
BACE1
180 kDa
130 kDa
100 kDa
70 kDa
55 kDa
40 kDa
35 kDa
25 kDa
BACE2
*
180 kDa
130 kDa
100 kDa
70 kDa
55 kDa
d
NRG1 III
180 kDa
130 kDa
100 kDa
70 kDa
55 kDa
40 kDa
35 kDa
NRG2ΔC
180 kDa
130 kDa
100 kDa
70 kDa
55 kDa
40 kDa
35 kDa
25 kDa

Figure 3

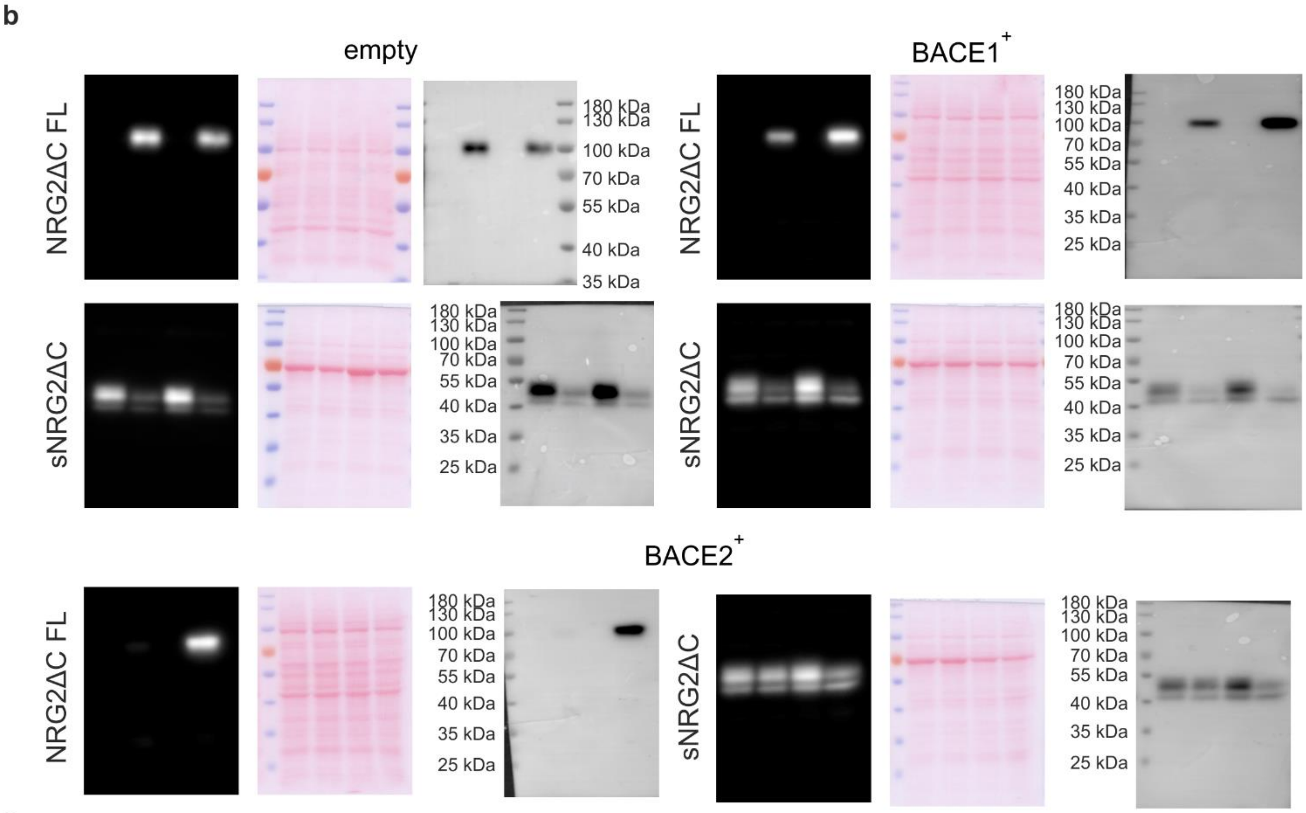
b
empty
BACE1+
NRG2ΔC FL
sNRG2ΔC
BACE2+
180 kDa
130 kDa
100 kDa
70 kDa
55 kDa
40 kDa
35 kDa
25 kDa

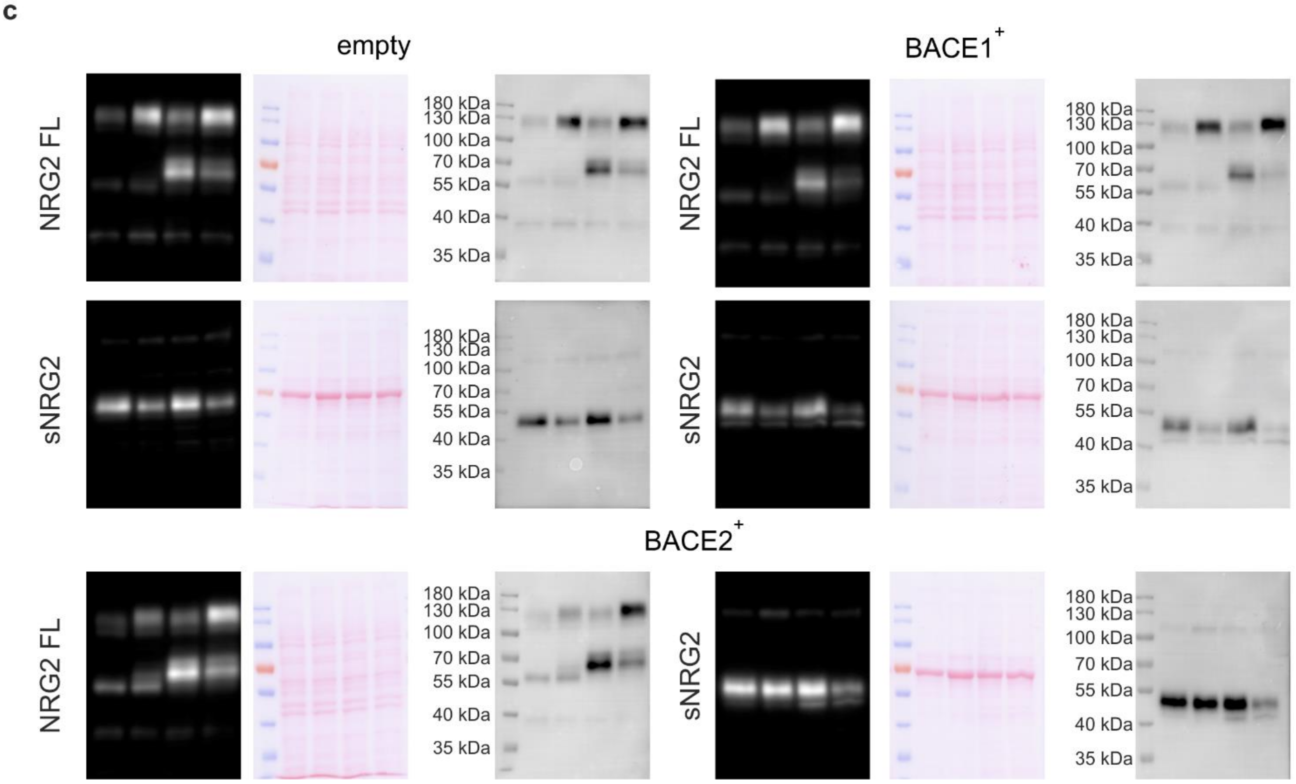
c
empty
BACE1+
NRG2 FL
sNRG2
BACE2+
180 kDa
130 kDa
100 kDa
70 kDa
55 kDa
40 kDa
35 kDa

Figure 4

a

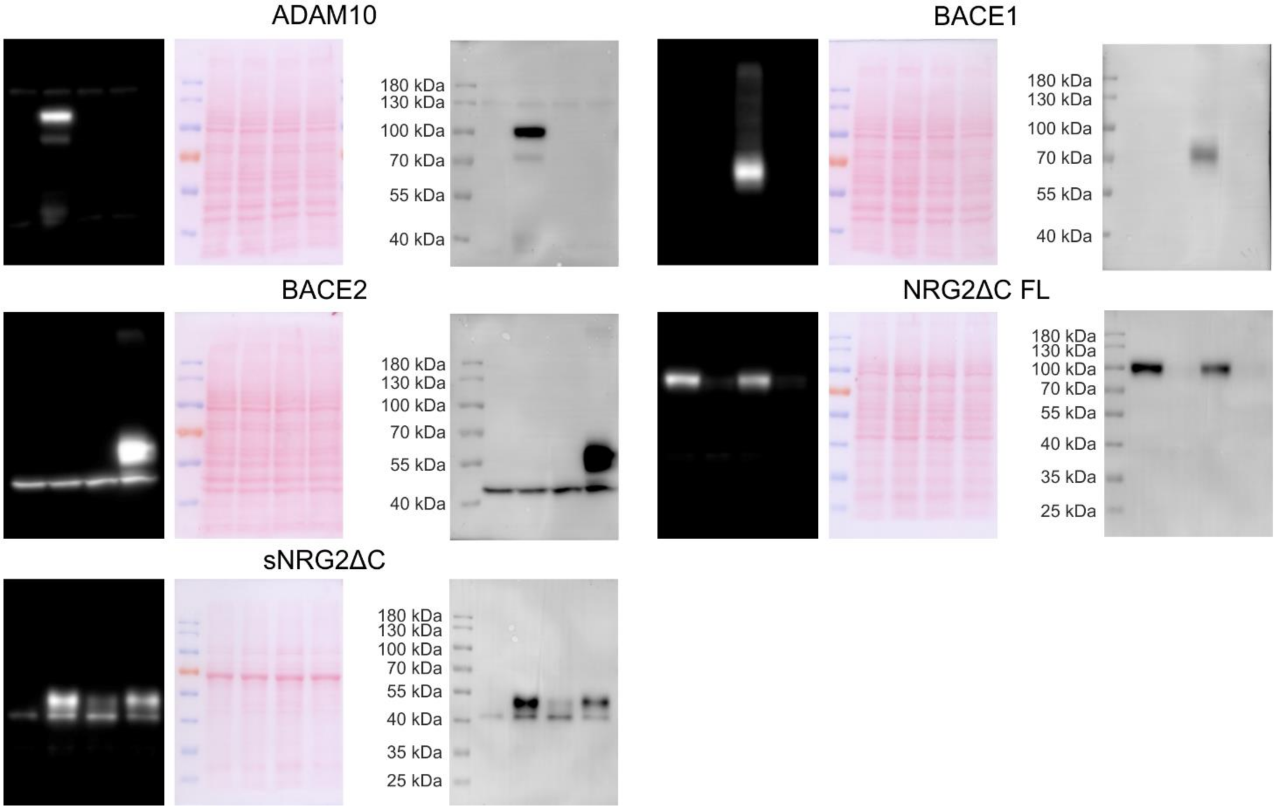

Figure 5

a

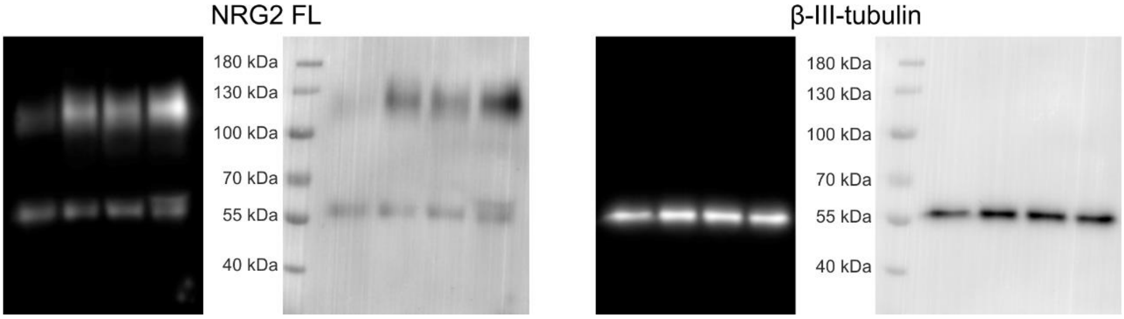

Figure 6

a

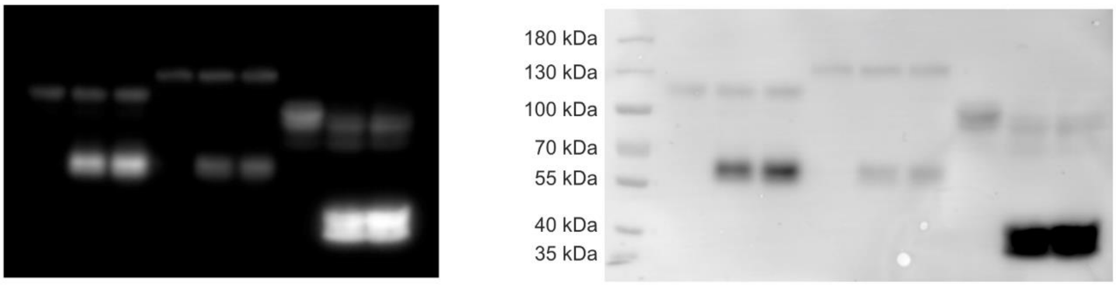

b

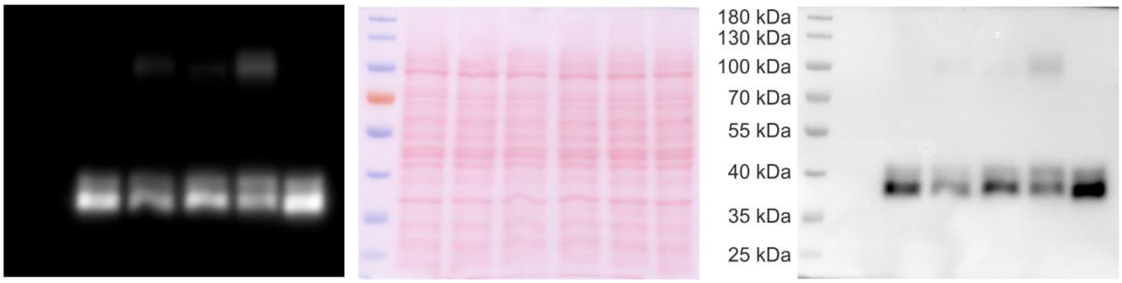

c

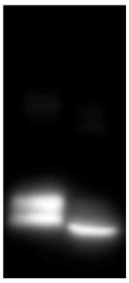

Figure 7

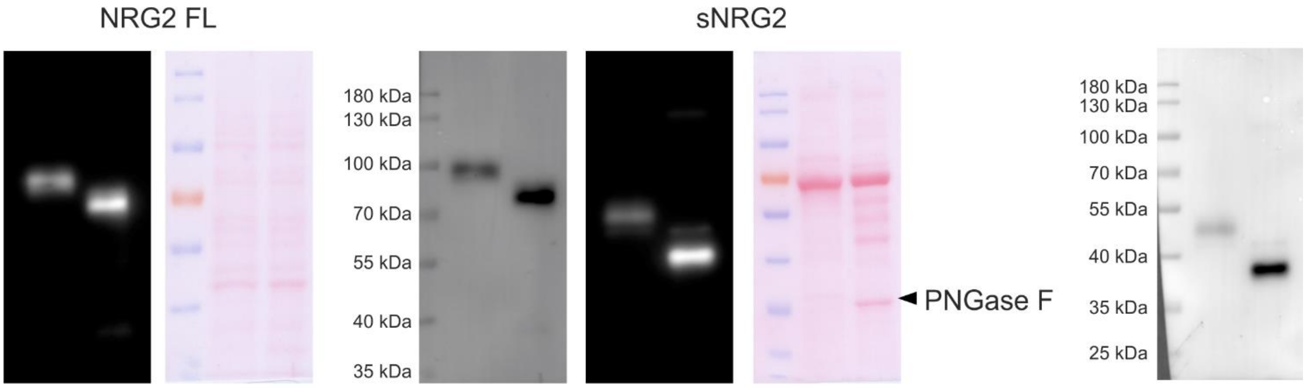

Supplementary Figure 1

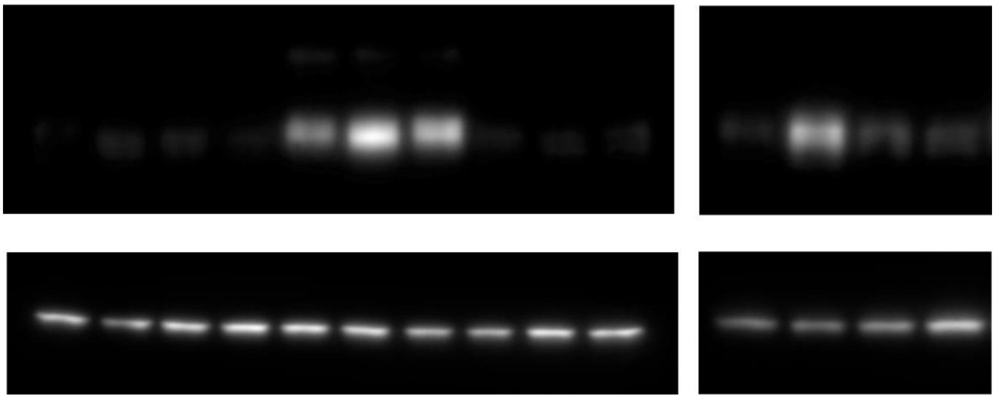

Supplementary Figure 3

a

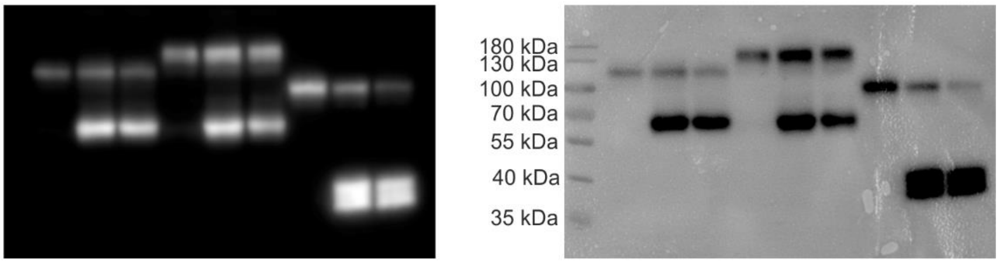

b

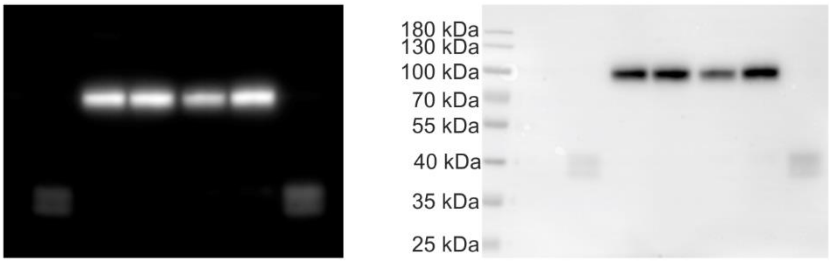

Supplementary Figure 4

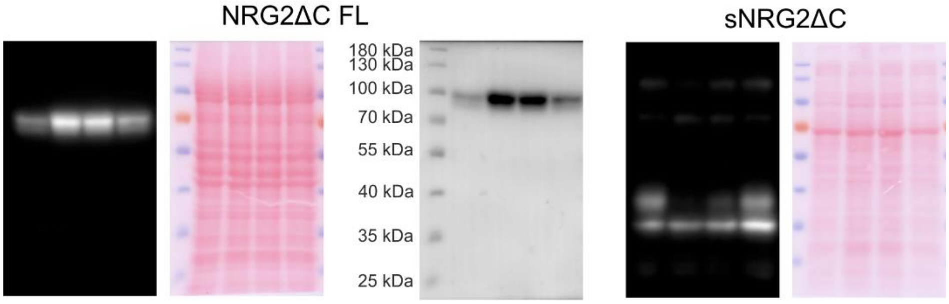

Supplementary Figure 5

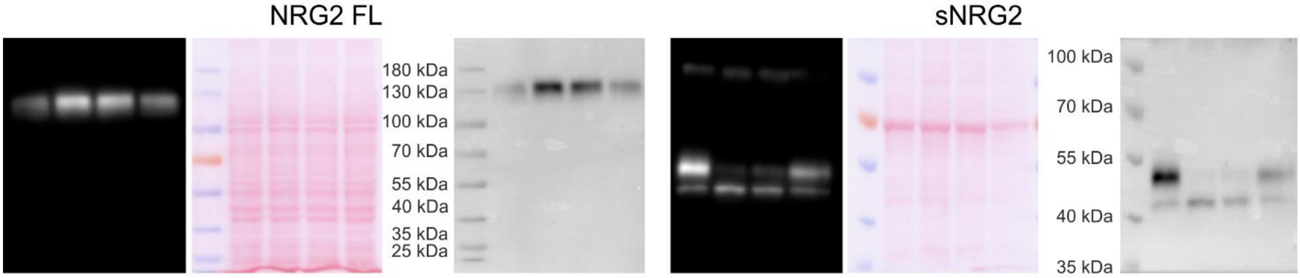

Supplement: Supplementary file 1 — (PDF 1631 kb) [file 12035_2019_1846_MOESM1_ESM.pdf]
